# Inhibition of Aurora Kinase Induces Endogenous Retroelements to Induce a Type I/III IFN Response via RIG-I

**DOI:** 10.1158/2767-9764.CRC-23-0432

**Published:** 2024-02-26

**Authors:** Lisa Choy, Stephen Norris, Xiumin Wu, Ganesh Kolumam, Ari Firestone, Jeffrey Settleman, David Stokoe

**Affiliations:** 1 Calico Life Sciences LLC, South San Francisco, California.

## Abstract

Type I IFN signaling is a crucial component of antiviral immunity that has been linked to promoting the efficacy of some chemotherapeutic drugs. We developed a reporter system in HCT116 cells that detects activation of the endogenous *IFI27* locus, an IFN target gene. We screened a library of annotated compounds in these cells and discovered Aurora kinase inhibitors (AURKi) as strong hits. Type I IFN signaling was found to be the most enriched gene signature after AURKi treatment in HCT116, and this signature was also strongly enriched in other colorectal cancer cell lines. The ability of AURKi to activate IFN in HCT116 was dependent on MAVS and RIG-I, but independent of STING, whose signaling is deficient in these cells. MAVS dependence was recapitulated in other colorectal cancer lines with STING pathway deficiency, whereas in cells with intact STING signaling, the STING pathway was required for IFN induction by AURKi. AURKis were found to induce expression of endogenous retroviruses (ERV). These ERVs were distinct from those induced by the DNA methyltransferase inhibitors (DNMTi), which can induce IFN signaling via ERV induction, suggesting a novel mechanism of action. The antitumor effect of alisertib in mice was accompanied by an induction of IFN expression in HCT116 or CT26 tumors. CT26 tumor growth inhibition by alisertib was absent in NSG mice versus wildtype (WT) mice, and tumors from WT mice with alisertib treatment showed increased in CD8^+^ T-cell infiltration, suggesting that antitumor efficacy of AURKi depends, at least in part, on an intact immune response.

**Significance:**

Some cancers deactivate STING signaling to avoid consequences of DNA damage from aberrant cell division. The surprising activation of MAVS/RIG-I signaling by AURKi might represent a vulnerability in STING signaling deficient cancers.

## Introduction

IFNs are cytokine mediators of the innate antiviral immune response. Type I IFNs (most commonly α or β), can be produced by most cells in response to viral infection. Type III IFNs (λ IFNs), are produced by surface-exposed epithelial cells and engage distinct receptors. However, inducers, downstream signaling and target gene expression patterns of type I and type III IFNs are largely identical. Major inducers of IFN expression include the pattern recognition receptor pathways that recognize signatures of viral infection such as inappropriately modified or localized DNA (e.g., cGAS/STING) or RNA (e.g., RIG-I/MDA5/MAVS; ref. 1). The role of IFNs is to orchestrate a suite of intracellular defenses against viral replication, increase class I MHC expression and antigen presentation, and stimulate production of cytokines such as CXCL10 that stimulate immune cell infiltration.

While immune checkpoint blockade (ICB) agents, such as those targeting PD-L1 or CTLA4 pathways, have demonstrated clinical efficacy in some cancers, current ICB treatments do not have activity in all cancers, sometimes due to an unfavorable tumor immune microenvironment (2). Triggering a local innate immune response in tumor cells could be one strategy to stimulate tumor immunogenicity. Indeed, the efficacy of many clinically approved small-molecule drugs is thought to be at least partially due to activation of local tumor-intrinsic immunity. For example, DNA methyltransferase inhibitors (DNMTi) have been shown to induce expression of suppressed endogenous retroelements (ERV), resulting in induction of IFN (3, 4), and expression of neoantigens (5, 6), both of which can stimulate immune responsiveness. Radiation and DNA-damaging chemotherapy have also been shown to promote tumor immunity via cGAS/STING-mediated induction of IFN (reviewed in ref. 7), which is sometimes accompanied by derepression of ERVs (8, 9). Thus, desilencing ERVs and triggering the activation of type I/III IFNs in tumor cells could represent a promising strategy to augment existing immune therapies.

Because IFN signal transduction is well characterized, several screens have been described taking advantage of well-defined STAT-binding DNA sequences driving artificial promoter-reporters to screen for activators of type I/III IFNs (10–16). Only one of these screens (13) tested responses longer than 24 hours. However, drugs that act through epigenetic or indirect mechanisms (such as DNMTis), could take longer than 24 hours to induce IFN, and might require interactions with chromatin of target genes in their native genomic context.

To identify novel activators of the innate immune response, we created a reporter cell line designed to capture inducers of the type I/III IFN pathway in the endogenous genomic context. We used it to screen a library of 1,443 well-annotated compounds, and discovered that Aurora kinase inhibitors (AURKi) were strong inducers of IFN. This activation was dependent on the innate RNA-sensing pathway components MAVS and RIG-I, and independent of STING in lines with cGAS deficiency, but dependent on STING in cells with wildtype (WT) STING signaling. Induction of ERVs was found to follow AURKi treatment. We also observed induction of IFN after AURKi administration in tumors grown in mice. The ability of AURKi to slow tumor growth depended partly on an intact immune compartment, suggesting that AURKi treatment augments antitumor immunity.

## Materials and Methods

### Reagents and Cell Lines

All cell lines were short tandem repeat–authenticated cell lines purchased directly from ATCC, expanded, and stock vials frozen down within two passages of receipt. Cells were grown in 10% FBS/RPMI, renewed from fresh stocks after 1 month in culture, and were confirmed negative for *Mycoplasma* monthly. Reagents for cell culture and lipid-based transfection were from Thermo Fisher Scientific. Reagents for nucleofection were from Lonza. Nucleic acid purification kits were from Qiagen. RNA sequencing (RNA-seq) library prep kits were from New England Biolabs. Oligonucleotides, Cas9 protein, guide cRNAs, and trans-activating CRISPR RNA (tracrRNAs) were from IDT, single-guide RNAs were from Synthego. Selective antibiotics were from Invivogen. IFNα (universal type I IFN) and IFNβ was from PBL Assay Science, IFNγ was from Sigma, and IFNλ2 was from R&D Systems. The drug library used for the screen (the “FDA-approved drug library” catalog L-1300) contained 1,443 different inhibitors and was from Selleckchem. Individual drugs were either from Selleckchem or MedChemExpress. Nocodazole was from Sigma.

### Cell Lines and Reporter Cell Line Generation

To create the *IFI27* reporter, we synthesized homology arms flanking the stop codon, either 814 bp upstream or 747 bp downstream of the stop codon, of the human *IFI27* gene (Genscript). We introduced a silent C→T mutation located 28 bp upstream of the *IFI27* STOP codon in the left homology arm, to destroy the PAM site following the guide sequence (bottom strand): CCTCGCAATGACAGCCGCAA. Between the homology regions, in place of the natural stop codon, a P2A self-cleaving peptide sequence (GSGATNFSLLKQAGDVEENPGP; ref. 17) was added, followed by the Clover *EGFP* gene with a C-terminal 3X SV40 nuclear localization signal, followed by a T2A self-cleaving peptide sequence (GSGEGRGSLLTCGDVEENPGP), followed by the nanoluciferase sequence, modified to contain the secretion leader peptide from the IL6 gene, and finally a new stop codon. Outside the right homology arm, we added the HSV-TK gene driven by a mini-TK promoter to enable negative selection against random integrants. The CRISPR RNA (crRNA) for the *IFI27* guide sequence (CCTCGCAATGACAGCCGCAA) was annealed with tracrRNA to comprise the two-component guide RNA (gRNA), and transfected along with the donor plasmid into an HCT116 cell line with stable Cas9 expression (18). The correctly integrated reporter was verified by PCR of genomic DNA and by RT-PCR for the hybrid *IFI27* transcript after IFNα treatment, then positive cells were enriched by 2 weeks 10 µg/mL ganciclovir selection. Cells were then treated 24 hours with IFNα and GFP+ cells isolated for single-cell cloning. The clone that had the highest induction of GFP/luciferase, and correct DNA sequence showing integration of the reporter into all copies of the *IFI27* locus, was used for all subsequent studies.

### Chemicals and Screening/Assay Procedures

The HCT116-*IFI27* reporter line was plated in 384-well plates, at 2,500 cells per well in 50 µL/well Fluorobrite DMEM with 10% FBS. The next day, drug library was added to a 2.5 µmol/L final concentration and cells were cultured for 7 days, then half the volume of cell supernatants was transferred to fresh plates for luciferase assay, while the remaining plate contents were subjected to Cell Titer Glo viability assay (Promega). For experiments with individual drugs, cells were incubated with drugs for 5 days prior to assay unless otherwise noted.

For the assay of conditioned medium from various cell lines using the HCT116-*IFI27* reporter, medium from drug-treated cells was diluted 50% and placed on the reporter cells for 24 hours, which is sufficient time to detect a direct IFN response, but not long enough for the residual drugs in the conditioned medium to themselves activate the reporter.

### RNAi and CRISPR

RNAi to Aurora kinase A and B was done using OTP smartpools from GE/Dharmacon, or using the individual siRNAs from these pools (Supplementary Table S1). The PPIB housekeeping gene was used as a negative control in some experiments, and the non-targeting control #2 was used in others. Transfections were done using RNAiMAX, with 10 nmol/L each siRNA or siRNA pool, and samples collected at 72 hours posttransfection.

CRISPR gene knockout (KO) was accomplished by lentiviral infection of guide expressing vectors in the HCT116-*IFI27* reporter cells, which stably express Cas9. The plasmid used for stable expression of gRNAs was described previously (18). Guide sequences were taken from the published Avana library sequences (19) or designed using the online CRISPOR tool Supplementary Table S1). In cell lines that did not have stable Cas9 expression, CRISPR KO was accomplished by transfection of RNP complexes as described (20). KO validation was done by Western blotting or by ICE analysis of PCR amplicons of target sites (Synthego; https://ice.synthego.com/#/).

### RNA, RNA-seq, and qPCR

RNA was extracted using the Qiagen RNeasy kit with DNAse digestion. For RNA-seq, libraries were prepared using the NEBNext Ultra II Directional RNA Library Prep Kit for Illumina. RNA-seq analysis was executed and visualized using an in-house, web-based platform, in which sequencing quality control was performed using FastQC (v0.11.5). Transcript expression was then quantified using Salmon (ref. 21; v0.9.1) in pseudoalignment mode, without adapter trimming, producing transcript-per-million (TPM) estimates, using GRCh38 for human reference transcriptomes. Differential expression analysis was performed in R using the Sleuth package (ref. 22; v0.29.0), producing gene-level effect sizes and Q-values for each comparison. The list of genes ranked by q-value was then used to perform gene set enrichment analysis (GSEA; refs. 23, 24). Hierarchical clustering and principal component analysis were also performed on the gene-by-sample matrix using built-in R functions. For qPCR, cDNA was prepared using the Bio-Rad iScript kit and targets amplified using Taqman Gene Expression 2X master mix along with the manufacturer recommended, predeveloped Taqman Gene Expression assay primer/probe mix (Thermo Fisher Scientific) for each target.

### Western Blotting, Flow Cytometry, and Immunofluorescence

For S10-H3 phosphorylation, HCT116-*IFI27* cells were treated with 200 ng/mL nocodazole and Aurora kinase inhibitors, and lysates prepared 24 hours later. For activity of individual Aurora siRNAs on Aurora kinase expression and phosphorylation, cells were transfected with siRNAs 72 hours prior to harvest, and treated as indicated with 200 ng/mL nocodazole 24 hours prior to harvest. Cell lysates were prepared in RIPA with protease/phosphatase inhibitors (Pierce) followed by sonication. Lysates were run on NuPAGE 4%–12% gels (Thermo Fisher Scientific) and transferred to nitrocellulose membranes. After 1–2 hours blocking, blots were probed overnight with primary antibodies (Supplementary Table S1). Secondary antibodies conjugated to fluorescent IR800 or IR680 (Licor) or horseradish peroxidase (Bio-Rad) were used at the manufacturer recommended dilutions.

To evaluate the EGFP response to IFN, HCT116-*IFI27* reporter cells were treated with IFNα for 24 hours. Cells were then harvested and analyzed on a Fortessa X-20 flow cytometer (BD).

For evaluation of γ-H2AX and micronuclei, HCT116-*IFI27* or HT29 cells were treated for 5 days with the indicated amounts of alisertib or barasertib, then fixed with 4% paraformaldehyde and stained with anti-phospho-H2AX Ser139 (Cell Signaling Technology) and anti-rabbit Alexa 568 secondary antibody, followed by Hoechst 33342 (both from Life Technologies). Cells were imaged using an Opera Phenix instrument using 100 ms exposure time and 20% power, and quantified using built-in Harmony software. The sum of means per nucleus were plotted for γ-H2AX and the total area of micronuclei per well were determined using the Harmony micronuclei detection function using the Hoechst channel.

### DNA Methylation Analysis

HCT116-*IFI27* reporter cells were plated at 2.3 × 10^5^/well of 6-well plates. The next day, drugs were added (alisertib 1 µmol/L, barasertib 100 nmol/L, or decitabine 100 nmol/L) or DMSO. Medium was changed and drugs refreshed at day 3. At day 5, cells were harvested and genomic DNA extracted with a Qiagen DNeasy kit. The Illumina Infinium MethylationEPIC array BeadChip (850K) and subsequent bioinformatics analysis was carried out by Epigenomic Services at Diagenode (catalog no. G02090000).

### *In Vivo* Studies

All animal studies were reviewed and approved by each institution's Institutional Animal Care and Use Committee prior to conduct. Care and use of animals was in accordance with the regulations of the Association for Assessment and Accreditation of Laboratory Animal Care. The HCT116 xenograft study was performed by WuXi. Athymic nude mice (Charles River Laboratories), ages 6–8 weeks, were implanted subcutaneously in the flank with 2 × 10^6^ HCT116 cells suspended 1:1 in matrigel. When tumors reached 100 mm^3^, mice were randomized to groups, and 10 were treated by intraperitoneal injection with 0.5 mg/kg decitabine, daily for 5 days, with a 2-day break, then daily for another 5 days. The remaining 20 mice were untreated for 5 days, then 10 mice were treated orally daily with vehicle alone (10% 2-hydroxypropyl-β-cyclodextrin and 1% sodium bicarbonate) while the other 10 were treated vehicle containing alisertib at 30 mg/kg, for 7 days. Tumors were measured every 2 days and mice weighed twice weekly. 1–2 hours after the final dose, mice were sacrificed, tumors flash frozen, and plasma collected for pharmacokinetic analysis.

For experiments with CT26 cells, BALB/cJ female mice, either WT or NSG (NOD.Cg-Prkdc^scid^Il2rg^tm1Wjl^/SzJ) strains, 8–10 weeks of age were used (Jackson Labs). CT26 cells were inoculated subcutaneously in the flank at 10^6^ cells per injection. Once tumor sizes reached an average of about 50 mm^3^, mice were randomized to the different groups, and dosing initiated with indicated doses of alisertib or decitabine, or vehicle only, by gavage daily. Tumors were measured with calipers and mice weighed twice weekly. At 14–16 days of treatment, tumors were harvested and flash-frozen or formalin-fixed for paraffin embedding. Tumor samples were sent to HistoBridge for embedding, sectioning, and CD8 staining and quantification.

### Statistical Analysis

*In vitro* experiments were performed at least three times and a representative experiment is shown. Data are presented as the means ± SDs. Two-tailed Student *t* tests using equal variance were performed in Excel, or for more complex datasets, two-way ANOVA in GraphPad Prism to assess significance between the comparisons indicated in each figure. Asterisks are used to indicate statistical significance [n.s., or no callout, statistically nonsignificant (*P* > 0.05); *, *P* < 0.05; **, *P* < 0.01; ***, *P* < 0.001].

### Data Availability

RNA-seq data will be made available upon request to the corresponding author.

## Results

### IFI27 Reporter Development and Screen

**FIGURE 1:**
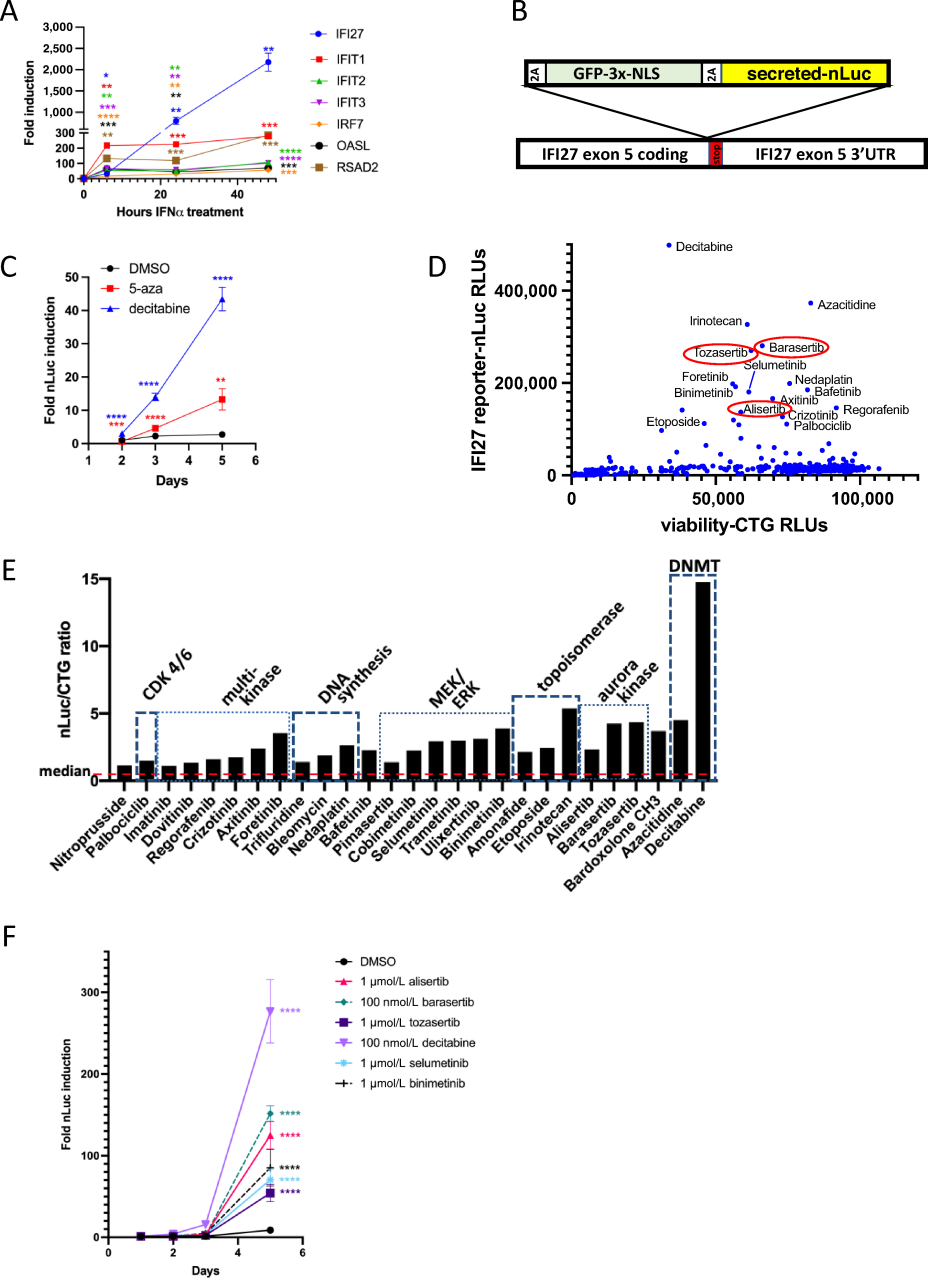
An endogenous IFN reporter generated at the *IFI27* locus was screened against an annotated drug library to identify Aurora kinase inhibitors as a novel hit. **A,** qPCR of canonical IFN target genes in HCT116 cells after 6, 24, or 48 hours after treatment with 1,000 U/mL IFNα. Significance shown for each gene at each timepoint versus the *t* = 0 value for that gene. **B,** Schematic of reporter construct. 2A = P2A or T2A skipper peptide sequence; NLS = SV40 nuclear localization signal; secreted = signal sequence from IL6 to cause export of nanoLuciferase from cells. **C,***IFI27* luciferase reporter induction after treatment with 2.5 µmol/L 5-azacytidine or 100 nmol/L decitabine for 2, 3, or 5 days. Significance shown for DMSO versus treatment for each timepoint. **D,** Raw nanoLuc and cell titer glo viability data from the *IFI27* reporter screen showing relationships between cell viability and reporter activation. Aurora kinase inhibitors are circled. **E,** Major active drugs from *IFI27* reporter screen, grouped by class and expressed as a ratio of nLuc/CTG. The median value of all compounds in the screen is overlaid as a red dashed line. **F,** Time course of reporter activation after alisertib treatment. Drugs were added as indicated and nanoluciferase activity of supernatants was assayed after the indicated number of days. Significance shown for DMSO versus treatment for each cell line at each timepoint (only day 5 achieved *P* < 0.05).

To create an endogenous type I IFN reporter, we chose the colorectal cancer cell line HCT116, which is highly efficient for CRISPR-based gene editing, and surveyed IFNα induction of canonical type I IFN target genes (*IFIT1*, *IFIT2*, *IFIT3*, *IFI27*, *IRF7*, *OASL*, *RSAD2*; Fig. 1A). We found that the *IFI27* gene was highly induced, with low baseline expression and high transcript levels after IFN treatment. RNA-seq confirmed *IFI27* is the second most highly induced IFNα response gene in HCT116 (Supplementary Table S2). We targeted the 3′ end of the *IFI27* coding region and inserted a nuclear-localized Clover GFP and a secreted nanoluciferase gene immediately before the stop codon (Fig. 1B). The reporter genes are separated from the *IFI27* gene by 2A sequences, allowing generation of intact proteins from a single transcript that retains all regulatory sequences intact.

Single-cell clones were verified for induction GFP and luciferase with IFNα treatment, then one clone, with genomic integration into all copies of the *IFI27* locus and similar morphology as the parent cell line, was selected for the subsequent experiments in this study. Induction of GFP could be detected starting at around 6 hours and was observed in most cells by 24 hours (Supplementary Fig. S1A–S1C). The IFNα response could also be measured by luciferase assays of cell supernatants and was completely inhibited by the JAK inhibitor ruxolitinib (Supplementary Fig. S1D). Induction of the reporter could also be seen in response to IFNβ and IFNλ2, a type III IFN (Supplementary Fig. S1D). Type II IFN could activate the reporter but was less effective, consistent with its reduced ability to induce the *IFI27* gene (Supplementary Fig. S1E).

Because DNMTis have been shown to activate IFN signaling (3, 4), we tested the *IFI27* reporter for DNMTi responsiveness. We found that the reporter was induced by DNMTis 5-azacytidine (5-Aza) and decitabine (Fig. 1C). Consistent with published reports and the mechanism of action of these drugs, which require cell division to incorporate into DNA, the induction of the reporter in response to DNMTis was slow relative to IFN treatment itself, barely detectable at 2 days but easily detectable by 5 days.

We treated our reporter cell line with a library of 1,443 well-annotated bioactive compounds and, to ensure sufficient time for epigenetic changes to elicit a phenotype, assayed after 7 days of treatment. The assay yielded a small number of hits that were not correlated with effects on viability (Fig. 1D). The strongest scoring hits fell into a few categories (Fig. 1E). Most of these drugs (inhibitors of DNMT, MEK, CDK, DNA synthesis, and topoisomerase) have been previously shown to induce type I/III IFN signaling (3, 4, 25–27), indicating that the screen worked properly. In addition, we saw reporter activation in response to inhibitors alisertib (modestly Aurora A selective), barasertib (Aurora B selective), and tozasertib (pan-Aurora). Kinetics of the reporter activation showed that, like with DNMTis, signal after AURKis or MEKis was low at 48 hours, and increased with time (Fig. 1F). The induction of IFN signaling by Aurora kinase inhibition had not, to our knowledge, been reported previously.

### Induction of IFN Depends on Inhibition of Aurora B

**FIGURE 2:**
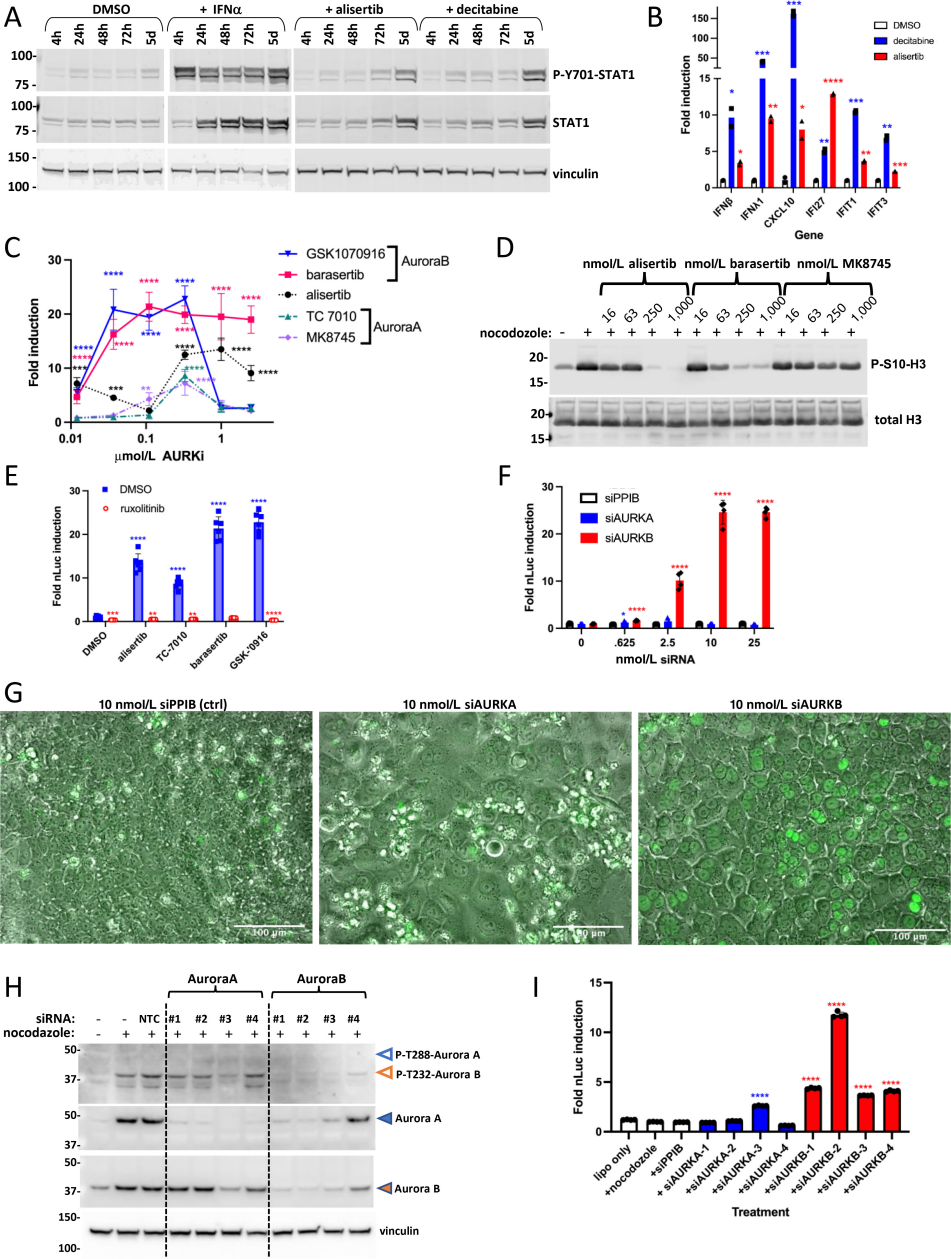
Activation of IFN is mediated through inhibition of Aurora B. **A,** Western blot analysis of the HCT116-*IFI27* reporter line treated with either DMSO, 1,000 U/mL IFNα, 1 µmol/L alisertib, or 100 nmol/L decitabine for the indicated amount of time. A total of 20 µg of whole cell lysates per lane were blotted with the indicated antibodies, with vinculin blotted as a loading control. **B,** qPCR analysis showing induction of IFN and target genes *IFNβ*, *IFNλ1*, *CXCL10*, *IFI27*, *IFIT1*, *IFIT3* after 5 days treatment of the HCT116-*IFI27* reporter line with DMSO, 100 nmol/L decitabine or 1 µmol/L alisertib. Significance is shown versus the DMSO control for each gene. **C,** Dose–response titration of Aurora A, Aurora B selective drugs on the *IFI27* reporter suggests that IFN induction occurs preferentially after inhibition of Aurora B. The Aurora A selective drugs TC-7010 and MK8745, the weakly Aurora A selective drug alisertib, and the Aurora B selective drugs barasertib and GSK1070916 were dosed at 12, 37, 110, 330, 1,000, or 2,500 nmol/L on the HCT116-*IFI27* reporter line for 5 days, then luciferase activity measured. Significance is shown for each drug versus the DMSO control at each timepoint. **D,** Western blot analysis of p-S10-H3 after treatment with alisertib, barasertib, or the Aurora A selective inhibitor MK8745 suggest that induction of the *IFI27* reporter coincides with inhibition of P-S10-H3, a biomarker of Aurora B activity. **E,** Ruxolitinib inhibition of *IFI27* reporter induction by Aurora kinase inhibitors demonstrates dependence of IFN induction by Aurora inhibition on JAK signaling. Cells were treated with DMSO, 1 µmol/L alisertib, 830 nmol/L TC-7010, 280 nmol/L barasertib, or 280 nmol/L GSK1070916, without or with 10 µmol/L ruxolitinib for 5 days, then luciferase activity measured. Significance shown for each treatment compared with DMSO alone. **F,** siRNA against Aurora A versus Aurora B shows that Aurora B inhibition is critical for induction of the IFN response. HCT116-*IFI27* reporter cells were transfected with siRNAs to PPIB, or pooled siRNAs (4 per target) to Aurora A or Aurora B, and luciferase activity was measured 5 days later. Significance is shown for each Aurora siRNA versus the PPIB siRNA condition for each dose. **G,** GFP fluorescent and phase imaging of cells shown in F, with characteristic morphologic changes accompanying siRNA transfection of either Aurora A or Aurora B, but induction of the reporter only with knockdown of Aurora B. **H,** Western blot analysis showing phospho-Aurora A/B and total Aurora A and B after siRNA knockdown. HCT116-*IFI27* reporter cells were transfected with the indicated siRNAs and 48 hours later additionally treated with nocodazole, then 24 hours later whole cell lysates prepared and blotted with Aurora A, Aurora B, or p-T288-Aurora A/T232-B antibodies. Vinculin blotting was also performed as a loading control. **I,** Individual siRNAs against Aurora A and Aurora B show that siRNAs against Aurora B activate *IFI27* reporter. HCT116-*IFI27* reporter cells were transfected with indicated siRNAs and reporter activity measured 72 hours later. Significance shown is relative to the siPPIB transfection.

To determine whether AURKis were activating IFN signaling more generally, we performed Western blot analysis to confirm STAT1 phosphorylation in HCT116-*IFI27* cells after alisertib treatment (Fig. 2A). This phosphorylation was similar in magnitude and kinetics to that induced by the positive control decitabine. Both compounds acted more slowly than direct treatment with IFN itself, as expected. To further confirm the activation of IFN signaling, we performed qPCR of canonical type I/III IFN target genes *IFI27*, *IFIT1*, *IFIT3*, and *CXCL10*, as well as *IFNβ*, and *IFNλ1*. We observed robust activation of all these target genes after alisertib treatment (Fig. 2B).

We tested Aurora isoform-selective compounds in the reporter line to assess the respective roles for Aurora kinase A or B in IFN signal induction. We found that Aurora B selective compounds were highly potent, whereas Aurora A selective compounds were less potent (Fig. 2C). Alisertib, which preferentially inhibits Aurora A, also inhibits Aurora B at higher doses (28), and activated the reporter to a similar extent as Aurora B selective compounds. The activity of higher doses of alisertib against Aurora B was confirmed by Western blotting showing inhibition of H3-S10 phosphorylation, an Aurora B target, by alisertib, but not by the highly Aurora A selective compound MK8745 (Fig. 2D). The effect of Aurora B inhibition on the reporter was blocked by cotreatment with the pan-JAK inhibitor ruxolitinib, confirming that IFN induction was responsible for reporter activation, not direct action of Aurora inhibition on the *IFI27* promoter (Fig. 2E).

As an orthogonal approach to targeting Aurora A versus Aurora B with small-molecule inhibitors, we also performed siRNA-mediated knockdowns. Transfected pooled siRNAs showed that activation of the reporter is associated with knockdown of Aurora B and not Aurora A (Fig. 2F and G), despite the induction of the enlarged cell phenotype typical of loss of either Aurora's function (Fig. 2G). As with Aurora small-molecule inhibitors, activation of the reporter by siRNA to Aurora B was inhibited by ruxolitinib (Supplementary Fig. S2). To better visualize Aurora kinases and activation-specific phosphorylation at the protein level, we transfected HCT116-*IFI27* cells with individual siRNAs against Aurora A or B, in the presence of nocodazole, which elevates AURK expression (29), and performed Western blot analysis. While both Aurora A and B were present, only Aurora B was detectably phosphorylated (Fig. 2H). Interestingly, knockdown of Aurora B also led to reduction of Aurora A. Whether this reduction is due to a dependence of expression of Aurora A on Aurora B, or cross-reactivity of the individual Aurora B siRNAs to the Aurora A sequence is not clear, although the sequence identity for each Aurora B siRNA for Aurora A is quite low (Supplementary Table S1). Reporter assays showed that the ability of individual Aurora siRNAs to induce IFN signaling corresponded to reduction in Aurora B protein (Fig. 2I). We conclude that the activation of IFN by AURKi requires Aurora B.

### IFN Signature Induction is Major Colorectal Cancer Response to AURKi

**FIGURE 3:**
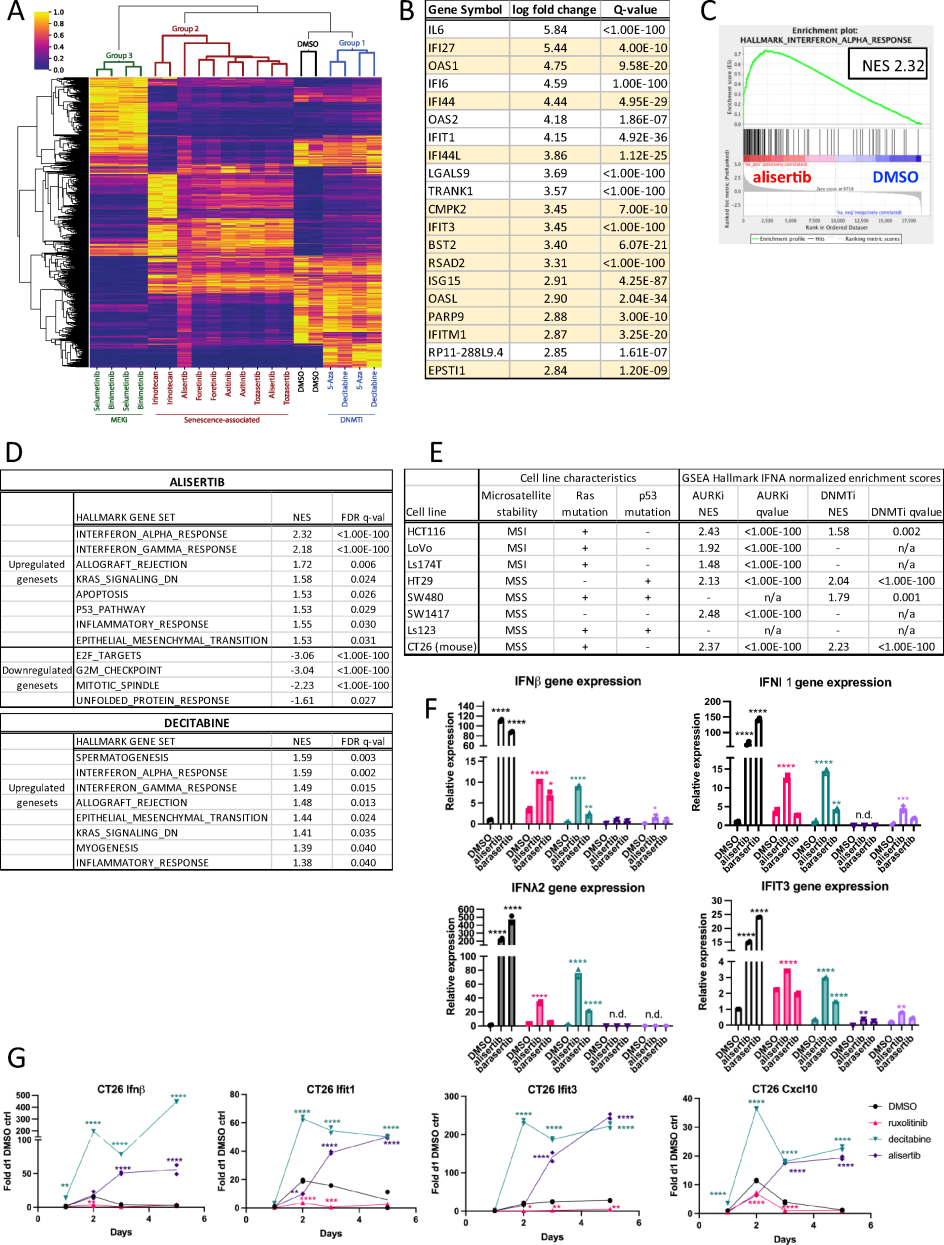
Aurora kinase inhibitors activate IFN signaling. **A,** Heat map of RNA-seq data from the HCT116-*IFI27* reporter line treated for 5 days with 100 nmol/L decitabine, 1 µmol/L 5-azacytidine, 1 µmol/L alisertib, 1 µmol/L tozasertib, 830 nmol/L foretinib, 2.5 µmol/L axitinib, 2.5 µmol/L irinotecan, 1 µmol/L selumetib, 830 nmol/L binimetinib, or an equivalent volume of DMSO. Heat map shows all significant genes using a *P*adj < 1e-10 in at least one condition. **B,** Top 20 most induced genes after alisertib treatment in the RNA-seq data shown in A, filtered for qval ≤ 0.05. Yellow colored genes are members of the Hallmark IFN Alpha gene set. **C,** GSEA hallmark IFN alpha gene set enrichment plot of RNA-seq data shown in A resulting from alisertib treatment. **D,** Summary of all positively or negatively enriched hallmark GSEA from the RNA-seq data after either alisertib or decitabine treatments, with a cutoff of FDR qval of <0.05. NES = normalized enrichment score. IFNα is the most highly induced of the enriched gene sets after AURK inhibition. **E,** Colorectal cancer cell lines were treated for 5 days with AURKi (1 µmol/L alisertib except for CT26, 1 µmol/L tozasertib) or the DNMTi decitabine (200 nmol/L), then RNA-seq performed and subjected to GSEA. GSEA normalized enrichment scores (NES) for the hallmark interferon alpha gene set are indicated along with the corresponding *q* values for each analysis. n/a = not applicable. **F,** qPCR measurement of expression of *IFNβ*, *IFNλ1*, and *IFNλ2* across the human colorectal cancer cell line panel after 5 days treatment with 1 µmol/L alisertib or 100 nmol/L barasertib. Values are relative to HT-29 with DMSO control treatment. Significance shown for each treatment versus DMSO for each cell line; n.d = not detected. **G,** CT26 cells were treated with DMSO, 10 µmol/L ruxolitinib, 1 µmol/L alisertib, or 200 nmol/L decitabine, and RNA collected at 24 hours, 48 hours, 72 hours, or 5 days. qPCR was performed to illustrate induction of Ifnβ and target genes Ifit1, Ifit2, and Cxcl10. Significance is shown for comparison between DMSO versus decitabine or alisertib for each timepoint.

To better characterize gene expression changes occurring in response to AURKis and selected other hits from the screen, we performed RNA-seq analysis of the reporter line after 5 days drug exposure (Supplementary Table S3). Gene expression patterns clustered into three groups (Fig. 3A). Inhibition of DNMTs by decitabine and 5-Aza represented Group 1; Aurora kinase inhibitors alisertib and tozasertib formed a second group that included other hits tested, foretinib, axitinib, and irinotecan; and MEK inhibitors binimetinib and selumetinib formed a third group. These results suggest that the IFN induction in response to AURKis may not be a direct effect of inhibiting Aurora kinase, but rather the state of the cells resulting from growth arrest or senescence. Cell morphology was concordant with the drug treatment categories identified within the RNA-seq analysis; cells that clustered with the AURKis in Group 2 were much bigger, with enlarged and abnormal looking nuclei compared with either DNMTi- (Group 1) or MEKi-treated (Group 3) cells (Supplementary Fig. S3A).

Many of the top genes induced by alisertib treatment were IFN pathway–related genes (Fig. 3B; Supplementary Table S3). GSEA of AURKi-treated cells revealed strong enrichment for the MSigDB hallmark IFNα response pathway (Fig. 3C); it is the most strongly enriched among all the hallmark enriched gene sets in both AURKi treatments (Fig. 3D; Supplementary Fig. S3B). This pathway is also enriched by decitabine (Fig. 3D), but less strongly than by AURKis. GSEA of the other drugs in Group 2, as well as Group 3, also showed strong enrichment of the hallmark IFNα pathway (Supplementary Fig. S3C–S3G).

To establish that IFN induction in response to alisertib is not limited to the reporter cell line, we performed RNA-seq on other colorectal cancer cell lines (LoVo, Ls174T, HT29, SW480, SW1417, and Ls123) treated with alisertib or decitabine (Fig. 3E; Supplementary Table S4). We observed that enrichment of the hallmark IFNα gene set was highly significant in most cell lines, and did not clearly track with microsatellite instability or Ras mutation status; although WT p53 was associated with all but HT-29. Within this panel, the IFN response is more frequently observed with AURKi than with DNMTi (Fig. 3E). In addition, we observed a modest (3-fold) increase in PD-L1 gene expression in HT29 which is consistent with a recent report on Aurora A kinase inhibition (30). However, we did not observe a significant change in PD-L1 expression in six of the seven cell lines tested (Supplementary Tables S3 and S4). We confirmed induction of IFN expression by qPCR of *IFNβ*, *IFNλ1*, *IFNλ2*, and *IFIT3* (Fig. 3F). We also found that the mouse syngeneic colorectal cancer cell line CT26 exhibited an IFN response to AURKi treatment (Fig. 3E and G).

**FIGURE 4:**
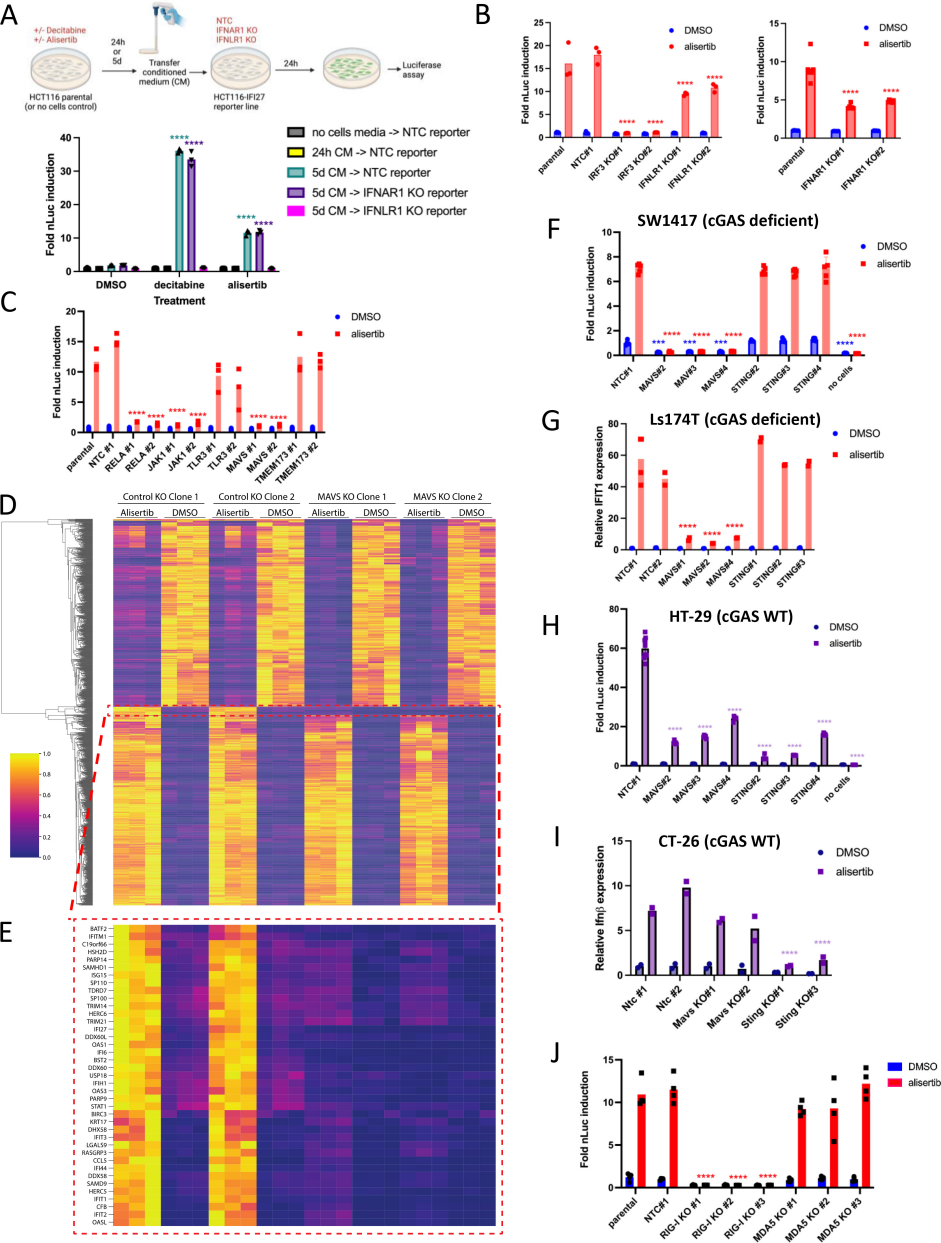
Aurora kinase inhibitors activate IFN signaling via secretion of IFN triggered via the MAVS/RIG-I pathway. **A,** HCT116-*IFI27* reporter cells secrete primarily type III but not type I IFN in response to DNTMi or AURKi. HCT116 cells were treated for 24 hours or 5 days with indicated drugs and the conditioned medium collected; alternatively, drugs diluted in culture medium were added to empty wells on the same plates. Conditioned media or drugs-only media were then applied to the HCT116-*IFI27* reporter line and assayed 24 hours later. Significance shown versus the DMSO control for each condition. **B,** Autocrine signaling in HCT116-*IFI27* cells is mediated by both type I and type III IFNs, and requires IRF3. HCT116-*IFI27* reporter cells were generated with CRISPR-mediated KOs of the indicated target genes. They were then treated with 1 µmol/L alisertib or an equivalent volume of DMSO, then luciferase activity at 5 days was normalized to the NTC cell line with DMSO treatment. Significance shown comparing each KO versus the parental with the same treatment. **C,** IFN induction by AURKi depends on JAK1, NFkB, and MAVS but not TLR3 or STING. HCT116-*IFI27* reporter cell lines with CRISPR KO of the indicated genes were treated for 5 days with 1 µmol/L alisertib, then luciferase activity measured and normalized to the NTC cell line with DMSO treatment. Significance shown comparing each KO versus the parental control for the same treatment. **D,** TPM heat maps for all significant genes (*P*adj < 1e-10 in at least one condition) after alisertib treatments in ctrl and MAVS KO clones. Group of genes that are differentially regulated in MAVS KO clones with alisertib treatment are boxed in red. **E,** Expanded view of red-boxed region of heat map in D showing IFN signature genes. **F,** IFN induction by alisertib in the cGAS-deficient line SW1417 depends on MAVS but not STING. NTC, MAVS, or STING KO SW1417 lines were treated with 1 µmol/L alisertib or DMSO, and the resulting conditioned medium, or drugs in media incubated without cells (“no cells”), was collected after 5 days. The conditioned medium was then overlaid 1:1 on the HCT116-*IFI27* reporter line, then luciferase activity measured 24 hours later. Values are relative to the NTC control with DMSO treatment and significance is shown for each genotype versus the NTC for the same treatment. **G,** IFN induction by alisertib in the cGAS-deficient line Ls174T depends on MAVS but not STING. NTC, MAVS, or STING KO lines were treated with 1 µmol/L alisertib or DMSO. After 5 days, IFIT1 transcript induction was assessed by qPCR and normalized to NTC#1 treated with DMSO. Values are relative to NTC#1 with DMSO and significance is shown for each genotype versus NTC#1 for the same treatment. **H,** IFN induction in cGAS-proficient lines HT-29 depends on STING more than MAVS. NTC, MAVS, or STING KO HT-29 lines were treated with 1 µmol/L alisertib or DMSO, and the resulting conditioned medium, or drugs in media incubated without cells (“no cells”), was collected after 5 days. The conditioned medium was then overlaid 1:1 on the HCT116-*IFI27* reporter line, then luciferase activity measured 24 hours later. Values are relative to the NTC with DMSO treatment and significance is shown for each genotype versus nontargeting control for the same treatment. **I,** IFN induction in cGAS-proficient lines CT26 depends on STING. Nontargeting, MAVS or STING KO CT26 lines were treated with 1 µmol/L alisertib or DMSO, and after 5 days, *Ifnβ* transcript induction was assessed by qPCR and expressed relative to the Ntc#1 treated with DMSO, and significance is shown for each genotype versus Ntc#1 for the same treatment. **J,** IFN induction by AURKi depends on RIG-I but not MDA5. HCT116-*IFI27* reporter cell lines with CRISPR KOs of RIG-I or MDA5 were treated for 5 days with 1 µmol/L alisertib or DMSO, then nanoluciferase activity measured. Values are relative to the NTC control with DMSO treatment and significance is shown for each genotype versus NTC#1 for the same treatment.

We next interrogated the signaling pathways required for this response. RNA expression data indicated that Aurora inhibition caused increases in *IFNλ1*, *IFNλ2*, and *IFNλ3* at the transcript level, as well as *IFNβ* (Fig. 2B; Supplementary Table S3). We were unable to detect IFNs in the conditioned medium of drug-treated cells using commercial ELISA kits. However, we were able to detect active IFN in conditioned medium from drug-treated parental HCT116 cells using the HCT116-*IFI27* reporter. To determine the contributions of type I versus type III IFN receptors to this signal, we generated HCT116-*IFI27* reporter lines with CRISPR KO of either *IFNAR1* or *IFNLR1*. Validated KO lines (Supplementary Fig. S4A) were then used to assay conditioned medium from drug-treated cells, to show that the activation of the reporter is dependent on IFNLR1 and independent of IFNAR1 (Fig. 4A). However, direct assay of AURKi induced *IFI27*-luc activity of *IFNAR1* or *IFNLR1* KO reporter cell lines themselves indicated KO of either IFNAR1 or IFNLR1 reduces reporter signal, suggesting both contribute (Fig. 4B). Because these cells do induce IFNβ at the transcript level, we speculate that the quantity of type I IFN in the conditioned medium may be too low to activate the reporter. We can conclude that active IFNs are made by cells in response to AURKi and they are responsible for *IFI27* reporter activation.

### IFN Response to AURKi Depends on MAVS/RIG-I Pathway

Previous studies have shown the STING pathway is required for inducing the IFN response to radiation (31), chemotherapeutic drugs (32), topoisomerase inhibitors (33), DNA mismatch repair deficiency (34), and senescence (35). Other studies have shown that the MAVS pathway mediates the IFN response to decitabine (3, 6), as well as irradiation (8). We observed morphologic changes, including micronuclei formation and increased γ-H2AX staining, in HCT116-*IFI27* (Supplementary Fig. S4B–S4F) and HT-29 (Supplementary Fig. S4G–S4K), in response to AURKi, suggesting possible involvement of DNA damage induced STING pathway activation. To explore which pathways may be required for AURKi to induce IFN, we made CRISPR KOs of *JAK1*, *RELA*, *IRF3*, *MAVS*, *TMEM173*, and *TLR3* in the HCT116-*IFI27* reporter cell line, verified knockdown of the target proteins (Supplementary Fig. S4L–S4Q) and measured the response to alisertib, as well as decitabine or IFNα for most lines. The reporter response to IFNα was dependent on JAK1, as expected (Supplementary Fig. S4R). The response to alisertib treatment was dependent on IRF3, JAK1, RELA, and MAVS, but independent of STING (Fig. 4B and C). In contrast, the response to decitabine was only partially dependent on JAK1, RELA, or MAVS, but also independent of STING (Supplementary Fig. S4S). This dependence on MAVS but not STING was recapitulated when using MAVS or STING KO single-cell clones rather than KO pools (Supplementary Fig. S4T and S4U). Barasertib, DNMTis, foretinib, and axitinib (other drugs from Group 2 in the RNA-seq study; Supplementary Fig. S4T–S4V) but not IFN (Supplementary Fig. S4W), were also dependent on MAVS and independent of STING. RNA-seq analysis examining the transcriptional response of MAVS KO clones to alisertib (Fig. 4D; Supplementary Table S5) revealed very few alisertib-mediated gene expression changes inhibited by MAVS KO (Fig. 4D, boxed). These were largely IFN target genes, and most of the genes from the MSigDB hallmark IFNα geneset that are induced by alisertib are no longer induced in the MAVS KO clones (Fig. 4E), suggesting that MAVS is required specifically for the IFN response, but not the overall response, to Aurora kinase inhibition.

The dependence of Aurora kinase inhibition on MAVS instead of the STING pathway to induce IFN is somewhat surprising, because Aurora kinase inhibitors have been documented to cause DNA damage (36), which has been reported to activate IFN via the cGAS/STING pathway (31–33, 37). While we demonstrated expression of STING in HCT116 when we validated the STING KO, we found that HCT116 cells failed to activate IFN signaling in response to STING agonist (Supplementary Fig. S4X and S4Y). Literature investigation revealed that HCT116 cells, as well as several other colorectal cancer lines, are reported to have deficiency in cGAS expression (38), and cannot activate STING. Our panel of colorectal cancer lines includes some lines with reported intact cGAS/STING signaling (HT-29, LoVo, CT26) and some with reported cGAS deficiency (HCT116, Ls174T, and SW1417; refs. 38, 39).

To determine whether the response to alisertib was MAVS dependent in colorectal cancer cells, we made CRISPR KOs of MAVS or STING in HT-29, CT26, Ls174T, and SW1417 cell lines (Supplementary Table S6) and assayed for IFN pathway induction in response to AURKis. We found that the cell lines with cGAS deficiency (SW1417, Ls174T; ref. 38) were dependent on MAVS for the IFN response (Fig. 4F and G), whereas cell lines with functioning cGAS/STING signaling (HT-29, CT26) exhibited STING dependency, but had reduced or no MAVS dependency (Fig. 4H and I).

MAVS is activated by binding to the cytosolic double-stranded RNA (dsRNA) sensor proteins RIG-I or MDA5, or the endosomal dsRNA sensor TLR3 (40). The IFN response to doxorubicin (41) and decitabine has been shown to depend on TLR3 (3) or by MDA5 and RIG-I (4), suggesting cellular context likely helps specify relative roles of the RNA-sensing pathways. Because we did not observe dependency on TLR3, we performed further CRISPR KO analysis to investigate the contributions of RIG-I and MDA5. We found the ability of alisertib to induce IFN signaling relied on RIG-I, but was independent of MDA-5 (Fig. 4J). Together, these data indicate that Aurora kinase inhibition induces IFN signaling by triggering the MAVS/RIG-I innate immune RNA-sensing pathway, which act through IRF3 and NFkB to induce type III (and to a lesser extent type I) IFN, which in turn activates the cognate IFN receptors to execute the IFN gene expression response via JAK1.

### AURKi Induces ERV Expression

**FIGURE 5:**
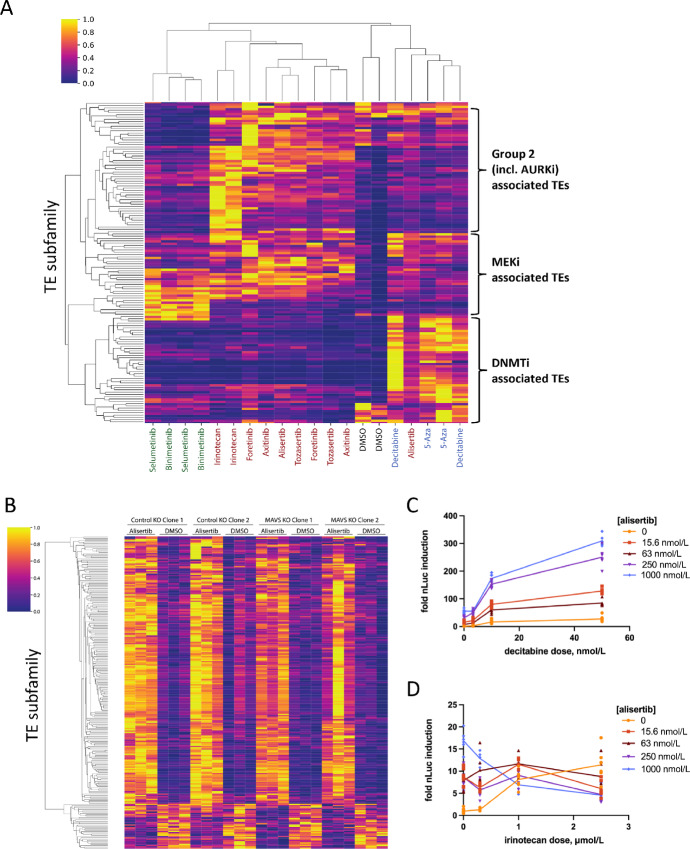
Induction of endogenous retroviruses by Aurora kinase inhibitors and screen hit drugs. **A,** TPM heat maps for all significant TEs, *P*adj < 0.05 in at least one condition after 5 days treatment with the indicated drugs as described in Fig. 3A. **B,** TPM heat maps for all significant TEs, *P*adj < 0.05 in at least one condition in MAVS KO or control clones after 5 days alisertib treatment. **C,** Synergistic interaction between AURKi and DNTMi for IFN induction. HCT116-*IFI27* cells were treated for 5 days with the indicated doses of alisertib and/or decitabine, then reporter luciferase activity was measured. **D,** No synergy between AURKi and irinotecan. As in C but with irinotecan instead of decitabine.

We next considered the source of AURKi-induced dsRNA that activates RIG-I/MAVS. One source might be ERVs, which have been shown to mediate the IFN response to decitabine treatment (3, 4), SETDB1 loss (42), and senescence (43). To determine whether ERV upregulation was associated with the induction of IFN by AURK inhibition, we used TEtranscripts (44) to quantify transposable element (TE) subfamily expression from our RNA-seq data (from Fig. 3C). As expected, DNMTis caused a strong upregulation of TEs, led by the HERV9-int subfamily (log_2_FC = 4.7 and 4.8 for 5-Aza and decitabine, respectively, see Supplementary Table S7). All Group 2 compound treatments, which includes the AURKis alisertib and tozasertib, broadly resulted in an upregulation of TEs, exemplified by the MER65C subfamily (log_2_FC > 1.9 in all Group 2 conditions). Interestingly, the three compound groups induced distinct subfamilies of TEs (Fig. 5A) where Group 2 compounds tended to cluster together, suggesting a conserved mechanism of TE upregulation shared across these compounds distinct from that of DNMTis. Next, we investigated whether upregulation of TEs by AURKis might be associated with loss of genomic DNA methylation, like DNMTis (3). We observed that decitabine treatment resulted in a profound loss of DNA methylation (*n* = 566,352 of 723,483 peaks with *P*adj < 0.05), while both alisertib and barasertib resulted in zero significantly (*P*adj < 0.05) methylated peaks (Supplementary Fig. S5A and S5B), consistent with a distinct mechanism. Importantly, alisertib resulted in similar TE upregulation in both MAVS KO cells and control clones even though the IFN signature was not triggered in the absence of MAVS (Fig. 5B). Thus, AURKis and other Group 2 drugs likely activate IFN signaling via derepression of retroelements in a manner distinct from loss of DNA methylation. Histone methylation, such as H3K9 and/or H3K27 is another established mechanism for ERV inhibition (45). We looked at global H3K9me3 and H3K27me3 levels after Aurora inhibition or siRNA, but were not able to detect reductions in the levels of these marks by Western that are consistent with IFN induction (Supplementary Fig. S5C and S5D).

To assess whether any well-characterized chromatin modifiers were systematically upregulated or downregulated after alisertib treatment, we reanalyzed our RNA-seq data with a focus on 429 well-characterized chromatin modifiers (46) in the five cell lines that showed an upregulation of IFN pathway genes (Supplementary Table S8). No genes scored as significant (*q* < 0.05) across all five cell lines, and only one (*IDH1*; downregulated upon treatment) scored as significantly differentially expressed across all cell lines with a more relaxed significance threshold (*q* < 0.1). Because loss of *IDH1* would drive an increase of DNA and histone methylation by elevating D-2-Hydroxyglutarate levels and thus promote silencing (47), we reason that this is unlikely to be the driving mechanism behind ERV desilencing upon alisertib treatment.

Because AURKis have a mechanism for activating ERVs that is distinct from DNMTis, we tested combination treatments in the HCT116-*IFI27* reporter. We observed strong synergy with decitabine (Fig. 5C; Supplementary Fig. S5E), whereas we observed either no or additive interaction between AURKis and DNA-damaging agents irinotecan (Fig. 5D; Supplementary Fig. S5F), etoposide, or 5-fluorouracil (Supplementary Fig. S5G–S5J). These data are consistent with AURKis acting on a similar pathway as DNA damaging agents but separate from DNMTis.

### AURKi Induces IFN *in vivo* and Depends on Immune Cell Function for Tumor Growth Inhibition

**FIGURE 6:**
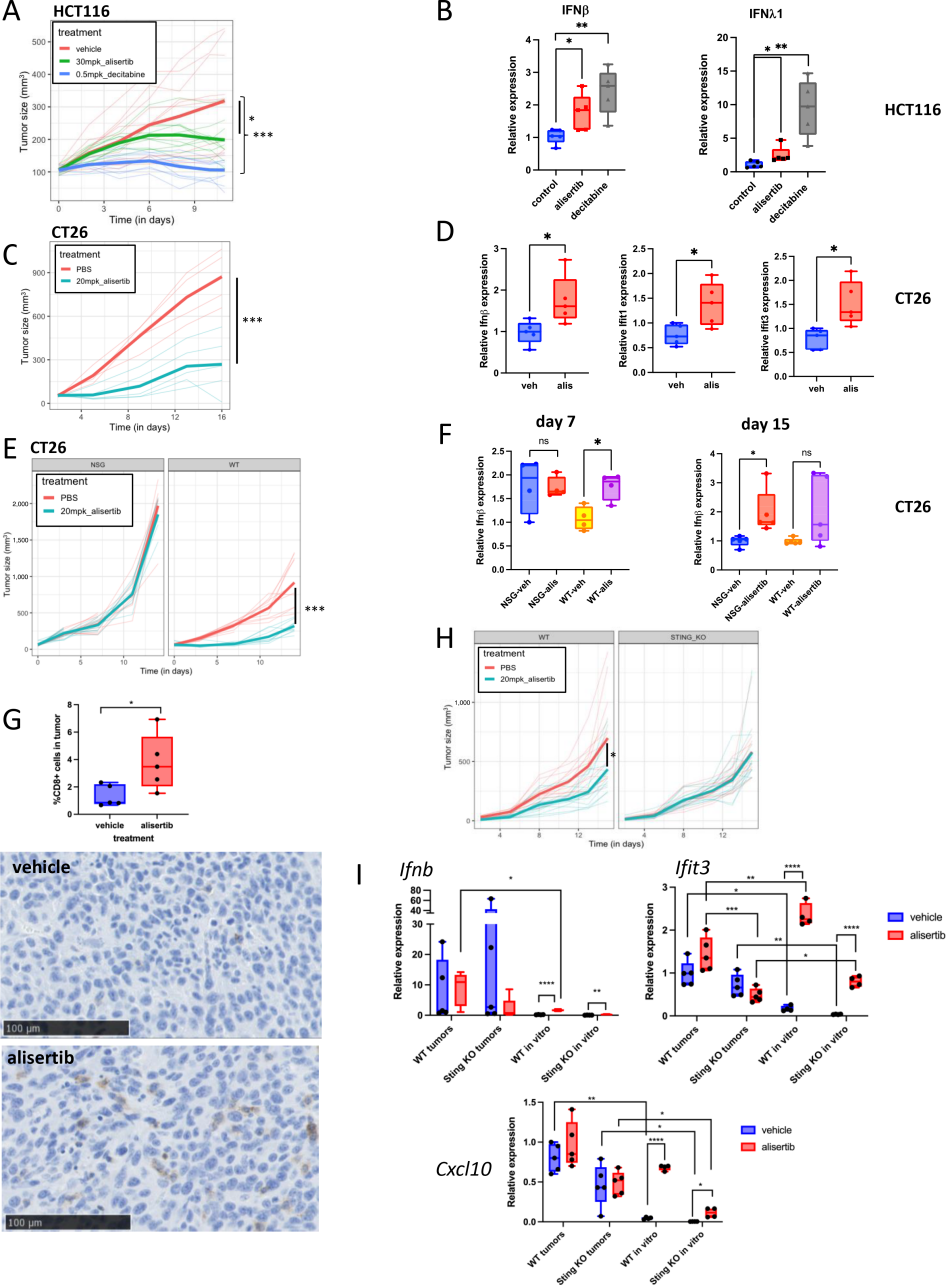
Alisertib induces IFN in tumors and depends on immune compartment and tumor-expressed Sting for efficacy *in vivo*. **A,** Alisertib and decitabine inhibit growth in HCT116 xenografts. Growth curves of HCT116 tumors in mice treated with vehicle, 30 mg/kg alisertib, or 0.5 mg/kg decitabine. **B,** IFN is induced in tumors after AURKi or DNMTi administration to mice. qPCR of *IFNβ* or *IFNλ1* from endpoint tumors from mice in A. **C,** Alisertib inhibits tumor growth of CT26 cells. Growth curves of CT26 tumors in mice treated with vehicle or 20 mg/kg alisertib, 5 mice per group. **D,** Alisertib administration induced IFN and IFN signature genes in CT26 tumors. qPCR of *Ifnβ*, *Ifit1* or *Ifit3* from endpoint tumors from mice in C. **E,** Alisertib inhibits growth of CT26 tumors in WT but not immune-deficient mice. Growth curves of CT26 tumors grown in NSG or WT Balb/c mice, 10 mice per group, treated with PBS vehicle or alisertib. **F,***Ifnβ* is induced by alisertib treatment of WT or immune-deficient mice. qPCR of *Ifnβ* from midpoint tumors (day 7) or endpoint tumors (day 15) from mice in E. **G,** Alisertib treatment causes an influx of CD8^+^ cells into CT26 tumors. Quantification and representative images of CD8 IHC from endpoint tumors from WT mice treated with vehicle or alisertib. **H,** Alisertib depends on Sting expression in CT26 cells to inhibit tumor growth. Growth curves of CT26 NTC control pooled clones or Sting KO pooled clones implanted into WT Balb/c mice, treated with 20 mg/kg alisertib or vehicle control (13 mice per group). **I,** Sting KO antagonizes the effects of alisertib on IFN signature genes. qPCR of *Ifnβ*, *Ifit3*, or *Cxcl10* from endpoint tumors from mice in H or from the same CT26 WT or Sting KO cell lines grown *in vitro* for 5 days with or without µmol/L alisertib.

We next wanted to determine whether AURKi induced IFN in tumors. Alisertib or decitabine inhibited tumor growth of HCT116 cells grown as xenografts (Fig. 6A). Both *IFNβ* and *IFNλ1* were significantly elevated in RNA prepared from tumors after alisertib administration, albeit to a lesser extent than after administration of decitabine (Fig. 6B). To address the consequences of IFN induction in the context of an intact immune system, we examined alisertib activity in a syngeneic model, CT26, which induces IFN signaling in response to alisertib treatment *in vitro* (Fig. 3E and G). Treatment of CT26 mice led to reduction of tumor growth (Fig. 6C) and increased expression of *Ifnβ* and IFN target gene expression (Fig. 6D).

To examine the dependency of alisertib on the adaptive immune system to affect tumor growth, we implanted CT26 cells into either WT Balb/c or NSG mice. We found that alisertib was ineffective at blocking tumor progression in the NSG mice, as opposed to WT mice (Fig. 6E). This result contrasts with results reported recently (30), but is consistent with another study (48). We could see a statistically significant induction of *Ifnβ* expression in either WT or NSG background after alisertib treatment, albeit at different timepoints (Fig. 6F). Levels of CD8^+^ T-cell infiltration of the tumors in WT hosts were found to be elevated in alisertib treated mice, consistent with alisertib and IFN induction, resulting in enhanced CD8^+^ T-cell recruitment (Fig. 6G).

CT26 cells induced IFN *in vitro* in a Sting-dependent manner (Fig. 4I). To determine whether Sting was required for the antitumor efficacy of alisertib and induction of IFN *in vivo*, we generated non-targeting control (NTC) control or Sting KO clones from CT26 cells (Supplementary Table S6), verifying Sting dependence of *Ifnβ* induction after alisertib treatment, and modest effects on cell viability (Supplementary Fig. S6A and S6B). When inoculated into WT mice, we confirmed that alisertib inhibited growth in NTC control tumors, but not in Sting KO tumors (Fig. 6H). Analysis of IFN pathway genes demonstrated elevated IFN signaling in tumor cells growing *in vivo* relative to *in vitro*. Moreover, a trend for increased IFN signaling in WT cells following alisertib treatment could be observed, which was not seen in the Sting KO cells (Fig. 6I). Taken together, the results suggest that Aurora kinase inhibition can activate IFN signaling *in vivo* and inhibit tumor growth in a manner at least partially dependent on the immune system and Sting signaling.

### Discussion

To find new activators of IFN signaling, we developed a screening platform that reports on the endogenous expression of a highly type I/III IFN activated gene, *IFI27*, and ran our screen for a sufficient time to capture epigenetic changes. Aurora kinase inhibitors were hits in our screen that had not previously reported to activate the IFN pathway. Indeed, one report (49) specifically tested whether AURKis could activate IFN, using a commercially available engineered cell line with an IFN response element driving a luciferase reporter, and observed no IFN induction response. This finding substantiates the utility of our CRISPR-based reporter, with its high degree of signal strength and structural authenticity that distinguish it from other IFN reporters.

RNA-seq analyses of the screen hits we tested revealed three general groups. DNMTis and MEKis formed distinct groups, while AURKis grouped with the other drugs tested: foretinib, axitinib, and irinotecan (“Group 2”). Group 2 drugs all generated an enlarged cell phenotype, and induce cell cycle arrest in the G_2_–M phase (50–53). MEKis arrest cells in G_1_ (54, 55) whereas DNMTis, at the doses we used, are not expected to cause significant changes in cell cycle distribution (56). Thus, the three expression profile groups are associated with distinct effects on cell cycle progression. The G_2_–M cell cycle arrest seen in Group 2 drugs are associated with defects in cell division that lead to DNA damage, aneuploidy and micronuclei (52, 57), which we observed (Supplementary Fig. S4C–S4F, S4H–S4K). Micronuclei can trigger cGAS-STING signaling (58); however, no direct connection between micronuclei and the RIG-I/MAVS pathway has yet been established. In contrast, DNA damage from irradiation has been shown to activate type I IFN signaling in a MAVS/RIG-I dependent manner; however, in this case, RIG-I was triggered by small noncoding RNA (sncRNAs) rather than ERVs (8). DNA demethylation cannot account for the induction of ERVs after AURKi treatment, and thus far we have not observed clear reductions in the global levels of ERV repression associated histone marks H3K9me3 or H3K27me3 that are consistent with induction of IFN. It is possible that such differences might become more obvious at the local chromatin level, and this question remains to be addressed. Currently, we speculate that the disordered state of cellular chromatin induced by treatment with Group 2 drugs, by interfering with DNA replication and cell division, results in failure to repress endogenous retroelements, and the compromised integrity of the nuclear membranes of G_2_–M arrested cells allows escape of these dsRNAs into the cytoplasm where they are detected by RIG-I.

The unexpected dependence of AURKis on the MAVS pathway may represent a novel vulnerability of tumors deficient in STING signaling. Indeed, some tumors have been shown to repress the cGAS/STING pathway as a means of immune escape (reviewed in ref. 37), and such tumors have been documented at a significant frequency in colorectal cancer (38). Therefore, we were interested to determine whether Aurora kinase inhibitors exhibited dependency on MAVS *in vivo*. However, none of the syngeneic models we examined (CT26, B16, 4T1, MC38, LLC) exhibited IFN induction in response to AURKi, or MAVS dependence (Fig. 4I; Supplementary Fig. S6C–S6F). We were able to confirm that AURKis could induce IFN *in vivo*, and that tumor growth inhibition by alisertib depended on immune system involvement, consistent with the hypothesis that AURKi-mediated IFN induction and ERV expression created a more favorable environment for immune recognition. Other investigators have also observed dependence of alisertib on the immune system to inhibit growth in syngeneic tumor models. Alisertib has been previously shown to increase CD8^+^ infiltration into CT26, MC38, and 4T1 syngeneic model tumors (48, 59).

The role of STING in the tumor compartment has been somewhat controversial. Some groups find that only the production of cGAS, but not the STING response, by the tumor cells is important; tumor-produced extracellular cGAS activates STING in immune cells in the tumor microenvironment (39, 60). Others found that STING signaling within the tumor compartment itself is required for inhibition of tumor growth in response to chemotherapeutics or radiation (31, 32). Perhaps STING signaling mediates antitumor effects in both tumor and immune compartments. In our study, we observed that STING expression in the tumor compartment was necessary for alisertib to inhibit tumor growth. STING expression also supported a higher level of IFN pathway activation in the tumors. Relieving this activation by deleting STING did not have a significant effect on tumor growth by itself. However, Sting was necessary for alisertib to exert tumor growth inhibition in this context.

The work described here shows that there are multiple mechanisms by which damaged cancer cells can activate the antiviral response and IFN signaling, not just the DNA-sensing pathway. The idea that our IFN reporter screen could identify drug candidates that may successfully combine with checkpoint blockade was substantiated by the approval of one of the hits from our screen for use in combination with immune checkpoint blockade (axitinib; KEYNOTE-426; NCT02853331). Additional screening with our reporter system could yield novel compounds to foster an immunogenic tumor microenvironment.

## Supplementary Material

Supplemental Table 1 Supplemental Table 1. Sequences of siRNAs, gRNAs, and antibodies used in this study.

Supplemental Table 2 Supplemental Table 2. RNAseq data from HCT116 cells treated 24h with 1000U/mlIFNalpha.

Supplemental Table 3 Supplemental Table 3. RNAseq data from drug treatments depicted in Figure 3A.

Supplemental Table 4 Supplemental Table 4. RNAseq from CRC lines.

Supplemental Table 5 Supplemental Table 5. NAseq from MAVS KO study.

Supplemental Table 6 Supplemental Table 6. ICE analysis of various CRISPR KO lines used in this study.

Supplemental Table 7 Supplemental Table 7. TEtranscripts analysis results from RNAseq data from Figure 3A.

Supplemental Table 8 Supplemental Table 8. Chromatin modifier gene expression data in CRC lines after alisertib treatment.

Supplemental Figure 1 Supplemental Figure 1. Validation of IFI27 reporter.

Supplemental Figure 2 Supplemental Figure 2. Inhibition of HCT116-IFI27 reporter induction by siRNA to AURKB by JAK inhibitor.

Supplemental Figure 3 Supplemental Figure 3. Morphologic and transcriptional changes in response to hits from IFI27 reporter FDA screen.

Supplemental Figure 4 Supplemental Figure 4. IFN/PRR pathway analysis.

Supplemental Figure 5 Supplemental Figure 5. Aurora kinase inhibition does not affect genomic DNA CpG methylation.

Supplemental Figure 6 Supplemental Figure 6. Characterization of CT26 Sting KO lines.

Supplementary Table Legends Supplementary Table Legends
