# Supplementary material for: Inhibition of Aurora Kinase Induces Endogenous Retroelements to Induce a Type I/III IFN Response via RIG-I
Source: Cancer Res Commun. 2024 Feb 26;4(2):540–55. doi: 10.1158/2767-9764.CRC-23-0432 (PMC10896070; doi:10.1158/2767-9764.CRC-23-0432)
Supplement: Supplemental Figure 1 — Validation of IFI27 reporter. [file crc-23-0432-s09.pdf]

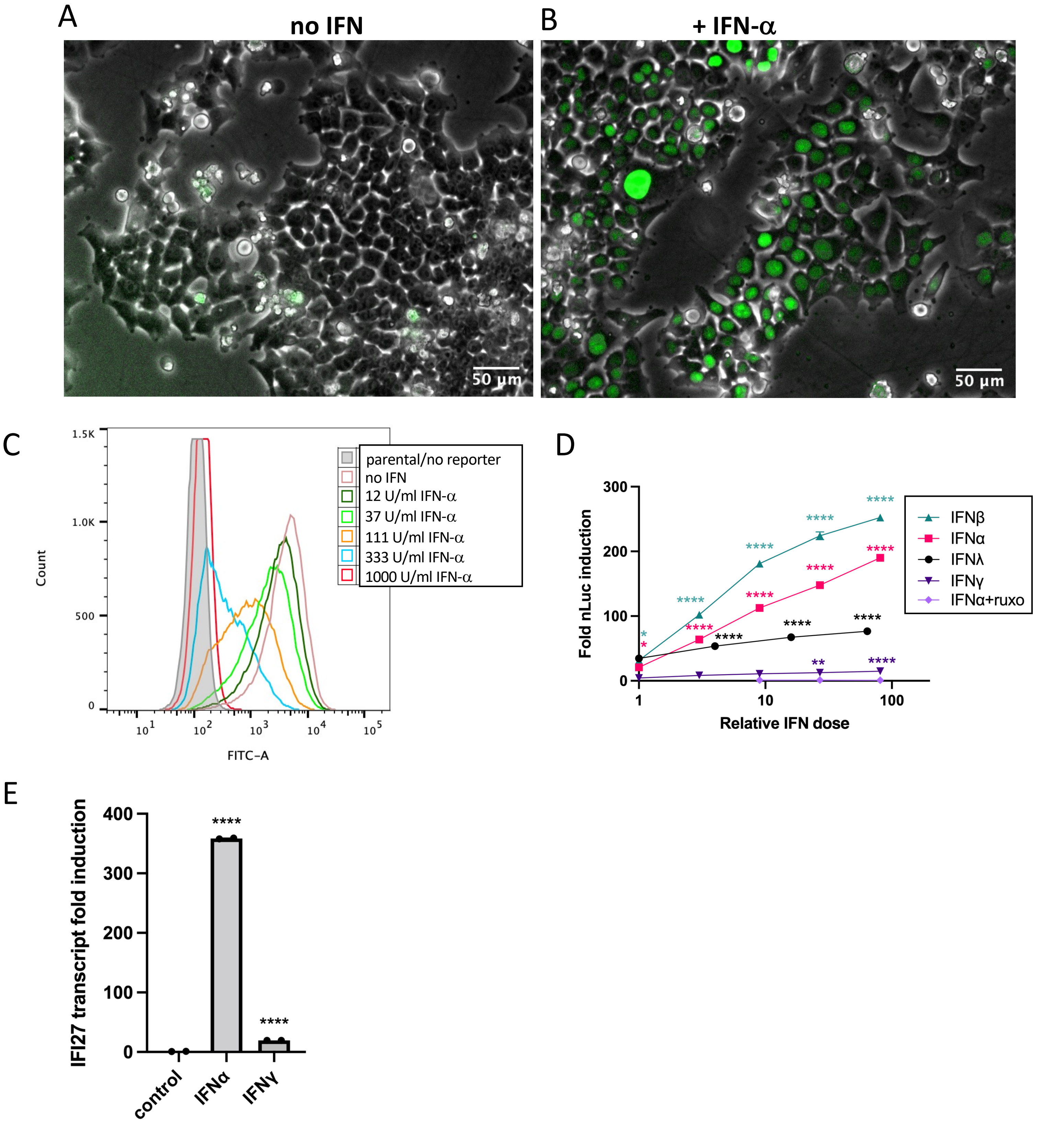

Supplemental Figure 1

Supplemental Figure 1. Validation of IFI27 reporter.

A) Phase/green fluorescence overlay of HCT116-*IFI27* reporter cell line, with no treatment.
B) Phase/green fluorescence overlay of HCT116-*IFI27* reporter cell line, with 1000U/ml IFNα, for 24h.
C) Flow cytometry showing increasing GFP signal after 24h with indicated doses of IFNα.
D) Dose-response of HCT116-*IFI27* reporter to type I, II, and III interferons. Luciferase after 24h treatment with IFNα, IFNβ (12, 37, 111, 333, or 1000U/ml), IFNλ2 (1.6, 6.3, 25, or 100 ng/ml), IFNγ (0.12, 0.37, 1.1, 3.3, or 10 ng/ml), or IFNα (111, 333, or 1000U/ml) + 10 µM ruxolitinib. Values here and in E) normalized to the untreated control reporter expression, and significance is shown for each IFN treatment vs the value of the untreated control.
E) Increase in HCT116-*IFI27* reporter is reflected in transcript levels. RNA was extracted from HCT116-*IFI27* reporter cells from control, 1000 U/ml IFNα or 10 ng/ml IFNγ for 24h and QPCR was performed to detect *IFI27* transcript levels.
