# Supplementary material for: Inhibition of Aurora Kinase Induces Endogenous Retroelements to Induce a Type I/III IFN Response via RIG-I
Source: Cancer Res Commun. 2024 Feb 26;4(2):540–55. doi: 10.1158/2767-9764.CRC-23-0432 (PMC10896070; doi:10.1158/2767-9764.CRC-23-0432)
Supplement: Supplemental Figure 2 — Inhibition of HCT116-IFI27 reporter induction by siRNA to AURKB by JAK inhibitor. [file crc-23-0432-s10.pdf]

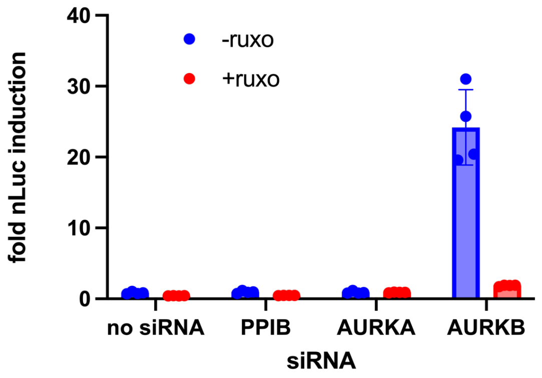

Supplemental Figure 2. Inhibition of HCT116-IFI27 reporter induction by siRNA to AURKB by JAK inhibitor. The reporter line was transfected with siRNAs against PPIB, AURKA, or AURKB, then ruxolitinib was added to cells at 10 µM final concentration ("+ruxo") or an equivalent volume of DMSO ("no ruxo").
