# Supplementary material for: Inhibition of Aurora Kinase Induces Endogenous Retroelements to Induce a Type I/III IFN Response via RIG-I
Source: Cancer Res Commun. 2024 Feb 26;4(2):540–55. doi: 10.1158/2767-9764.CRC-23-0432 (PMC10896070; doi:10.1158/2767-9764.CRC-23-0432)
Supplement: Supplemental Figure 3 — Morphologic and transcriptional changes in response to hits from IFI27 reporter FDA screen. [file crc-23-0432-s11.pdf]

A

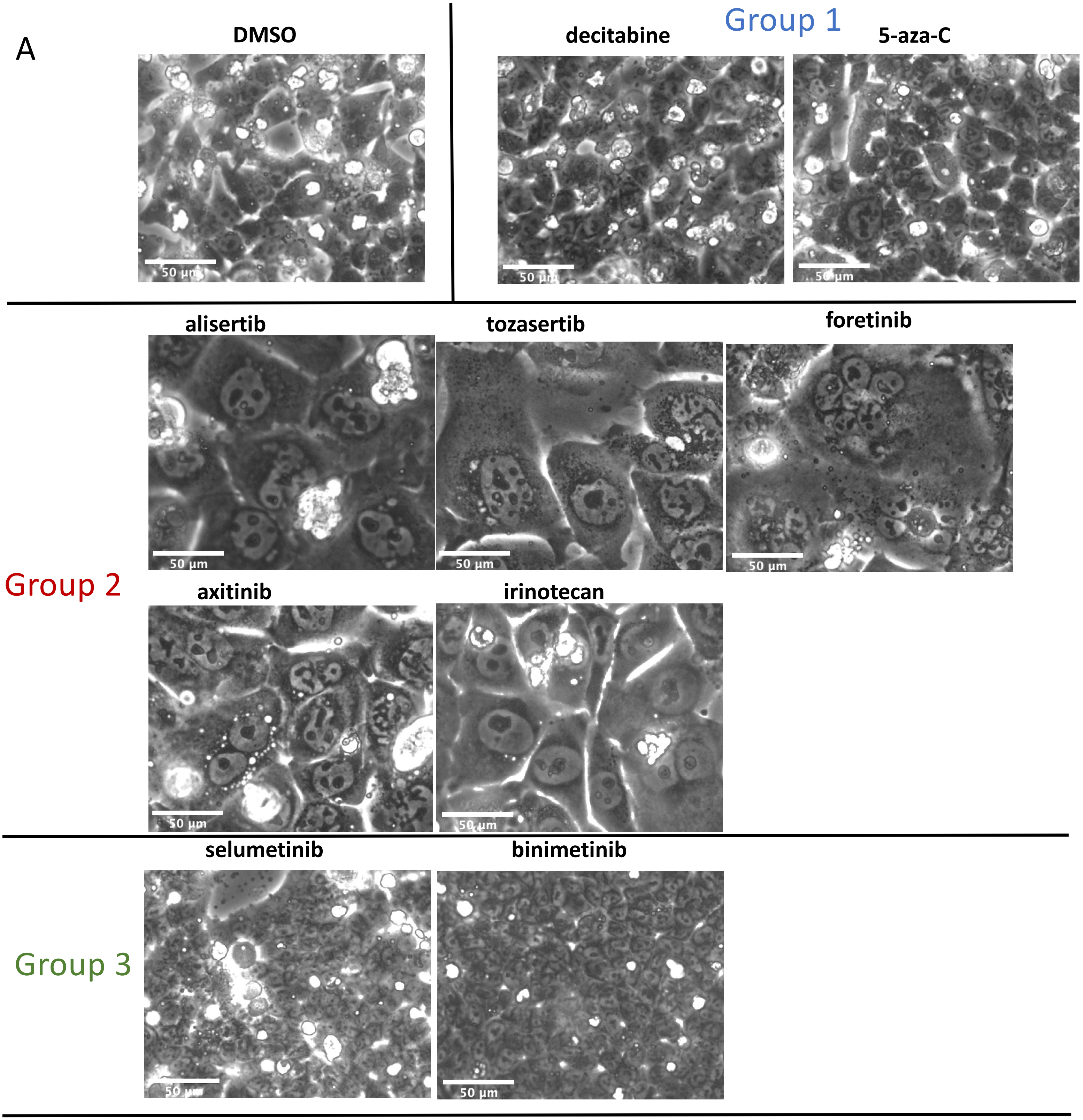

B

TOZASERTIB			
	HALLMARK GENE SET	NES	FDR q-val
upregulated genesets	INTERFERON_ALPHA_RESPONSE	2.33	<1.00E-100
	INTERFERON_GAMMA_RESPONSE	2.20	<1.00E-100
	P53_PATHWAY	1.66	0.013
	OXIDATIVE_PHOSPHORYLATION	1.58	0.024
	ALLOGRAFT_REJECTION	1.50	0.042
downregulated genesets	G2M_CHECKPOINT	-2.62	0.000
	E2F_TARGETS	-2.52	0.000
	MITOTIC_SPINDLE	-2.00	<1.00E-100
	KRAS_SIGNALING_UP	-1.72	0.009
	SPERMATOGENESIS	-1.70	0.009
	UNFOLDED_PROTEIN_RESPONSE	-1.68	0.011
	TNFA_SIGNALING_VIA_NFKB	-1.66	0.011
	PANCREAS_BETA_CELLS	-1.62	0.014
	ESTROGEN_RESPONSE_LATE	-1.59	0.017
	IL2_STAT5_SIGNALING	-1.58	0.016
	EPITHELIAL_MESENCHYMAL_TRANSITION	-1.47	0.045

Supplemental Figure 3

C *axitinib treatment*

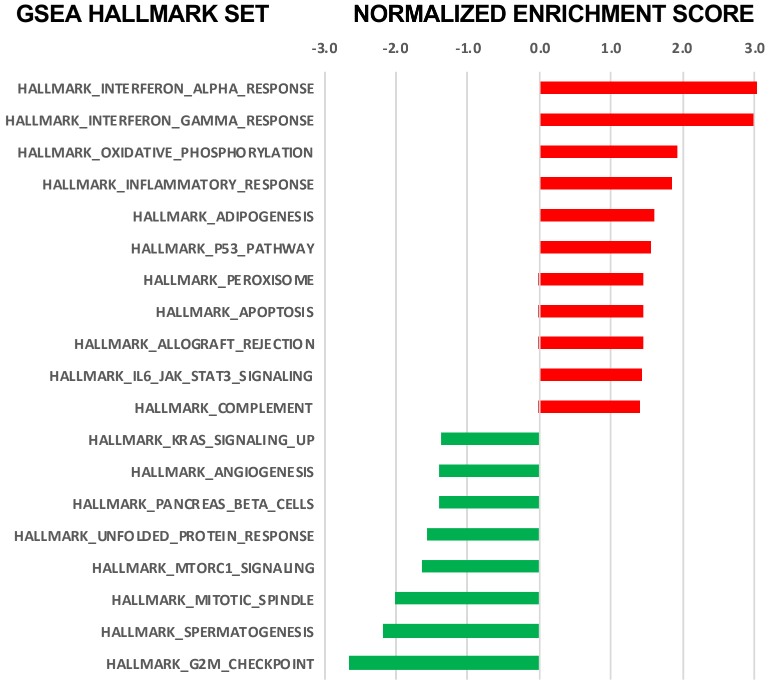

D *irinotecan treatment*

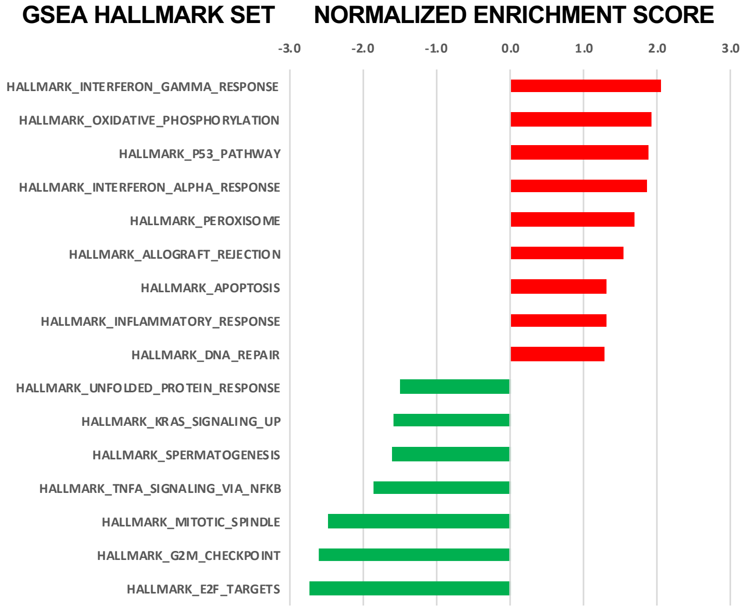

E *foretinib treatment*

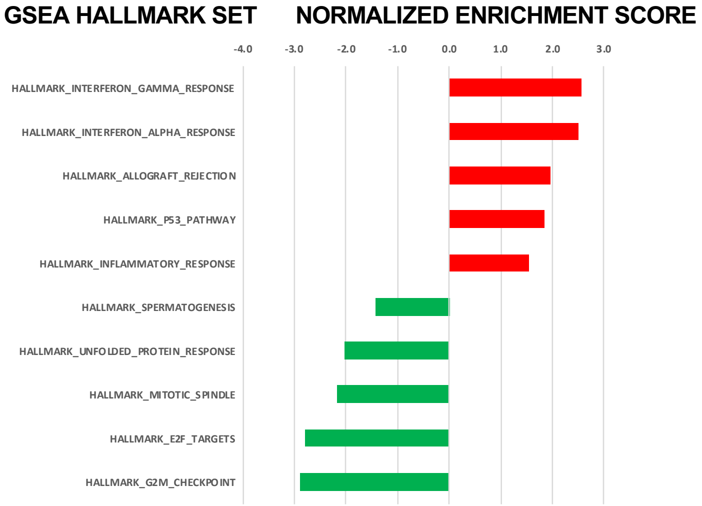

F *binimetinib treatment*

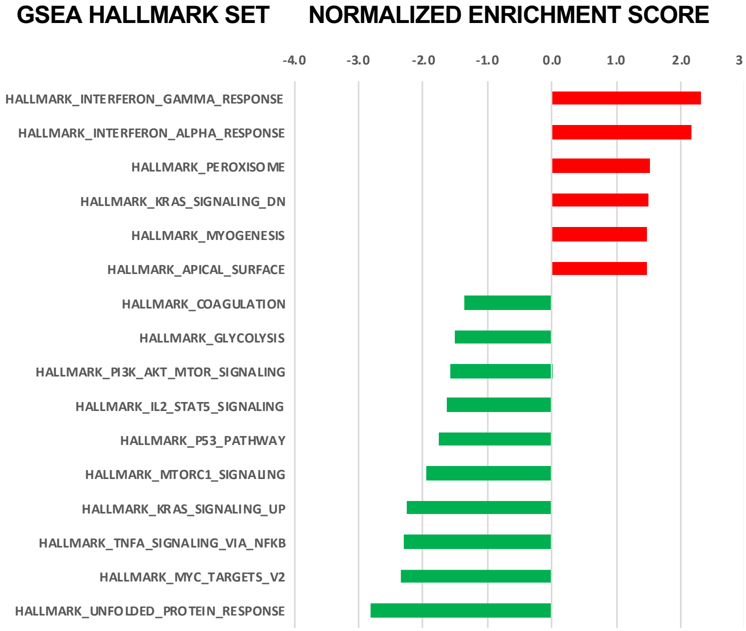

G *selumetinib treatment*

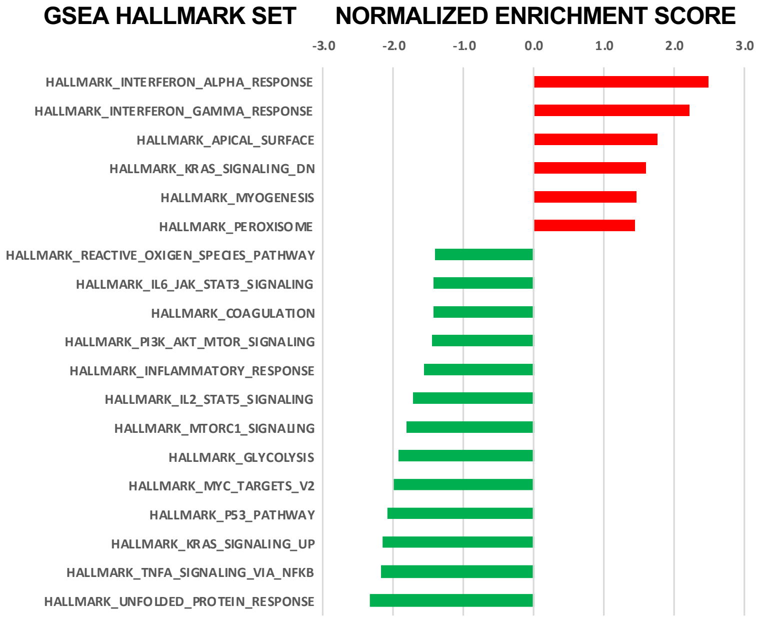

Supplemental Figure 3, cont'd

Supplemental Figure 3. Morphologic and transcriptional changes in response to hits from *IFI27* reporter FDA screen.

A) Images of drug treated cells used for RNAseq analysis in Figure 3A) showing the cell morphologies and their similarities in accordance with clustering by gene expression.
B) Summary of all positively or negatively enriched hallmark GSEA data sets with a cutoff of FDR qval of <.05, after 5 days treatment with 1 μM tozasertib. NES = normalized enrichment score. Interferon alpha is the most highly induced of the enriched gene sets after AURK inhibition.
C) Similar to B) but after 2.5 μM axitinib treatment.
D) Similar to B) but after 2.5 μM irinotecan treatment.
E) Similar to B) but after 830 nM foretinib treatment.
F) Similar to B) but after 830 nM binimetinib treatment.
G) Similar to B) but after 1 μM selumetinib treatment.
