# Supplementary material for: Inhibition of Aurora Kinase Induces Endogenous Retroelements to Induce a Type I/III IFN Response via RIG-I
Source: Cancer Res Commun. 2024 Feb 26;4(2):540–55. doi: 10.1158/2767-9764.CRC-23-0432 (PMC10896070; doi:10.1158/2767-9764.CRC-23-0432)
Supplement: Supplemental Figure 4 — IFN/PRR pathway analysis. [file crc-23-0432-s12.pdf]

A

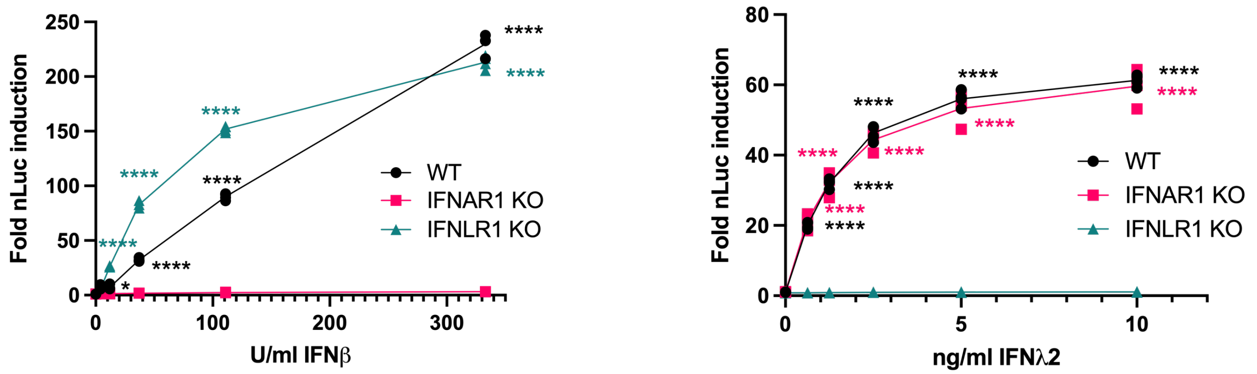

Supplemental Figure 4

B

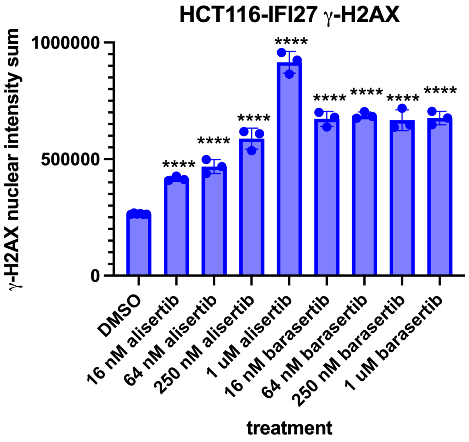

C

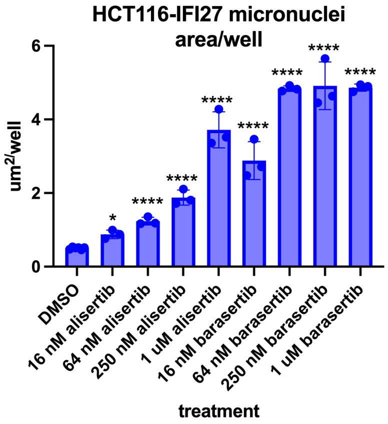

D HCT116-IFI27 - DMSO

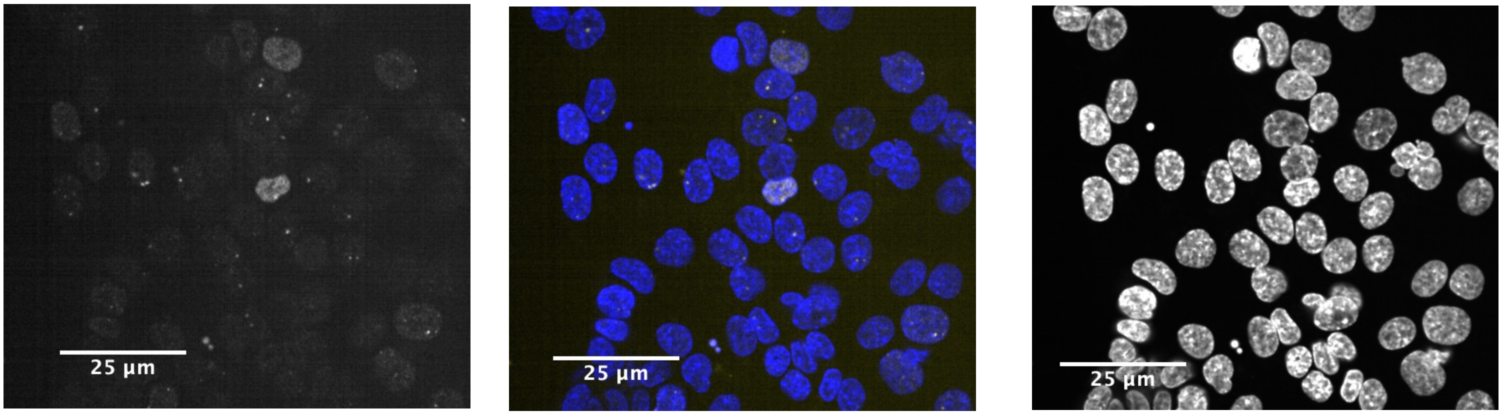

E HCT116-IFI27 – 1 μM alisertib

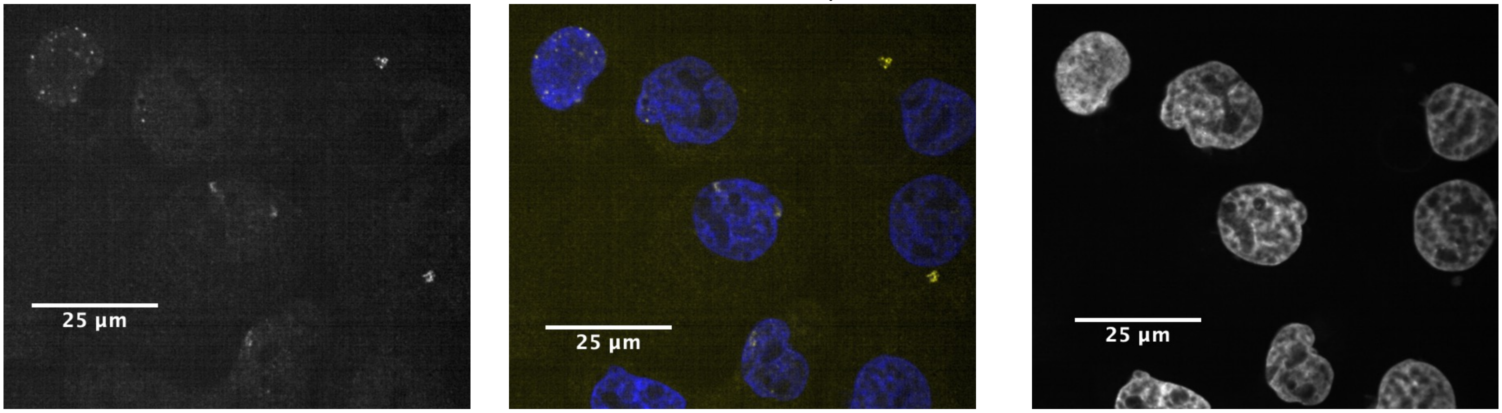

F HT116-IFI27 – 250 nM barasertib

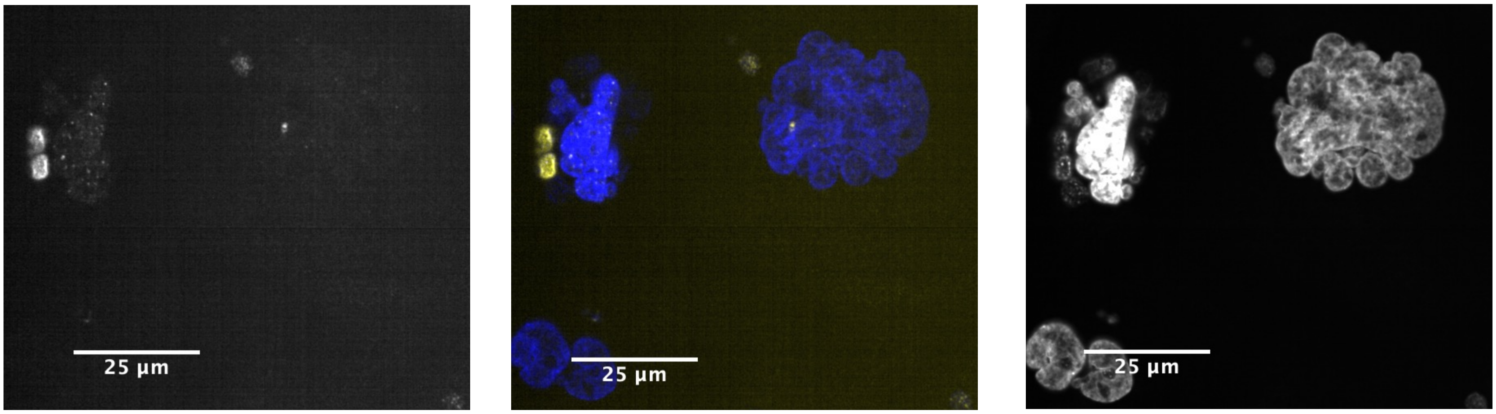

γ-H2AX merge Hoechst 33342

Supplemental Figure 4 cont'd

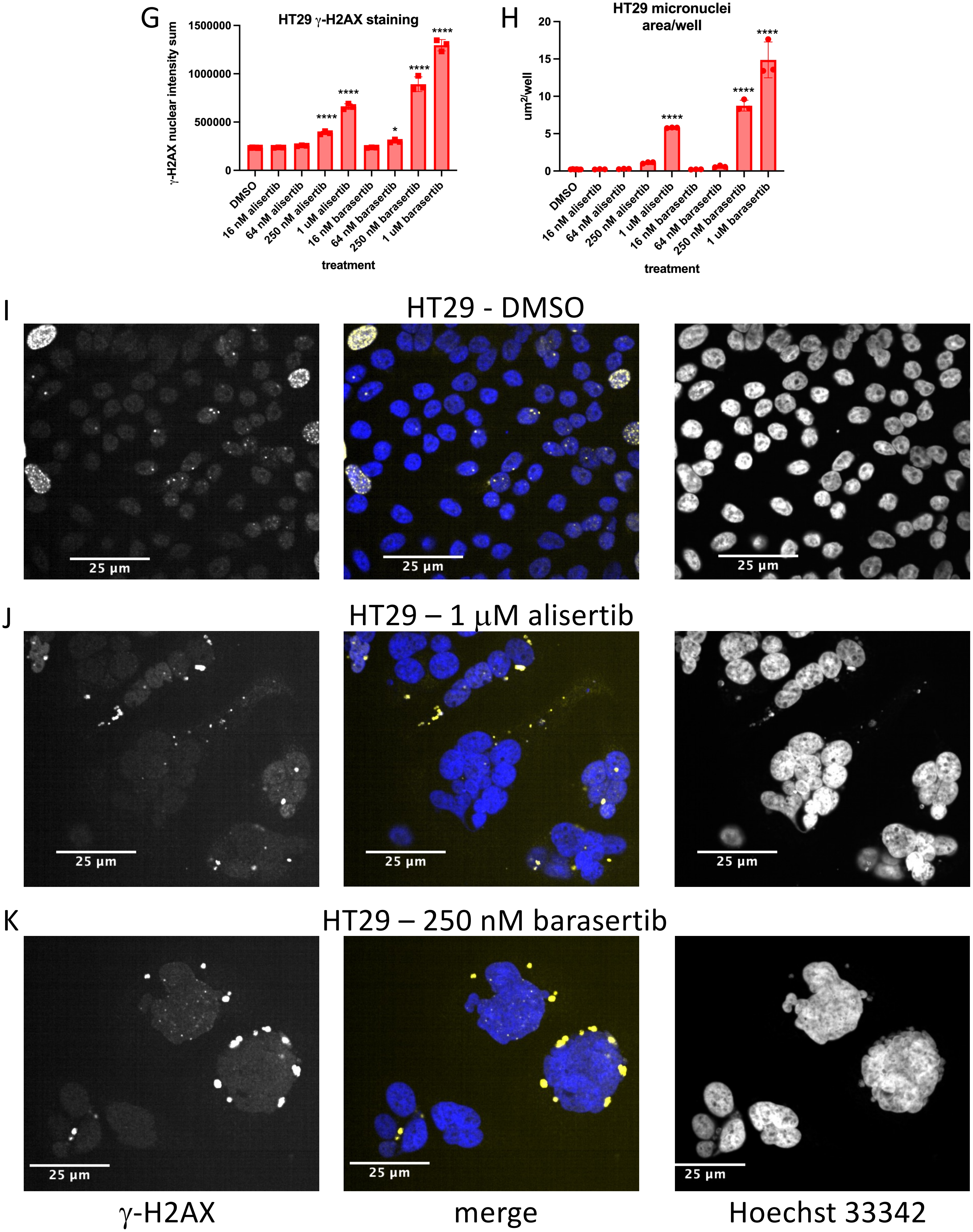

Supplemental Figure 4 cont'd

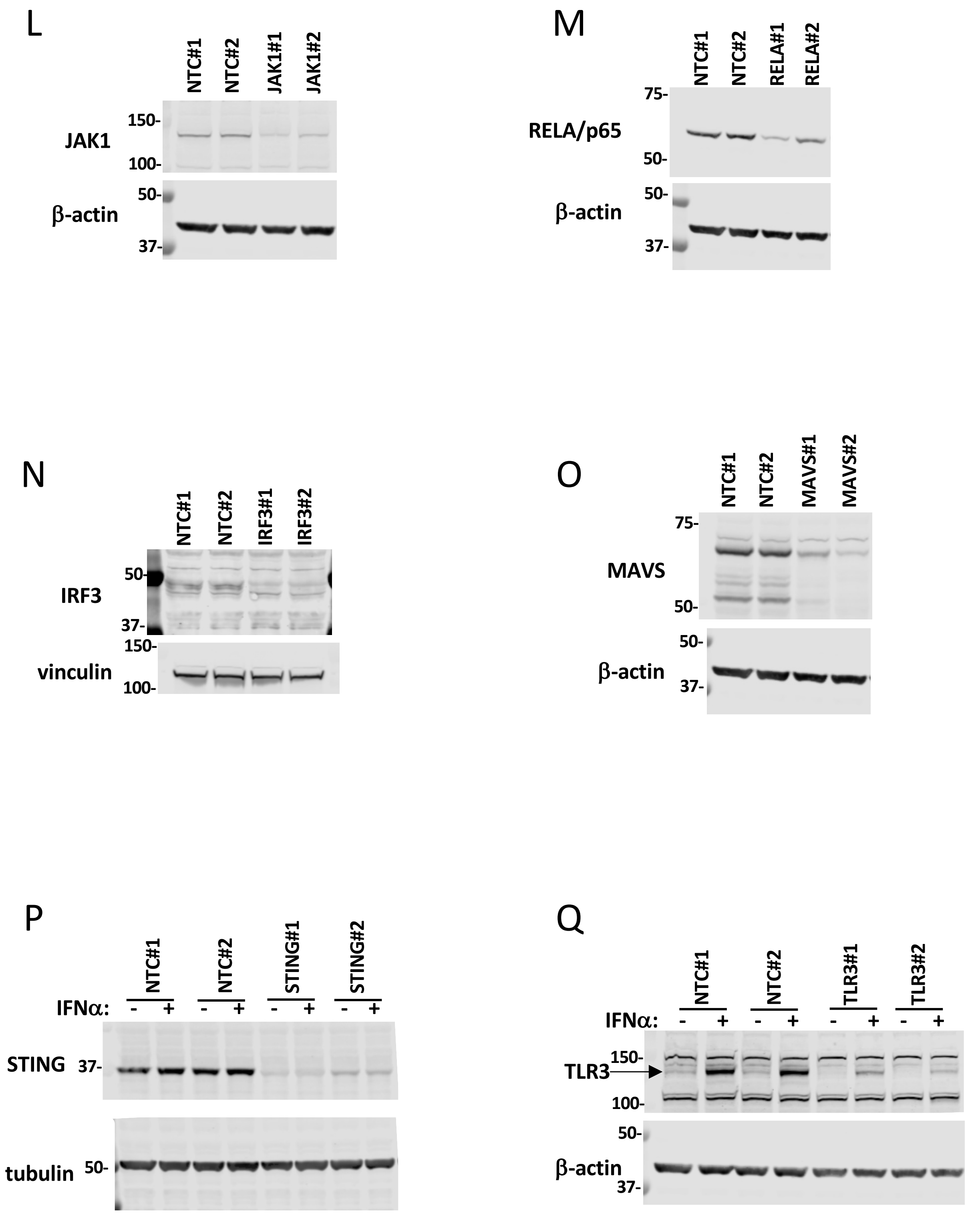

Supplemental Figure 4 cont'd

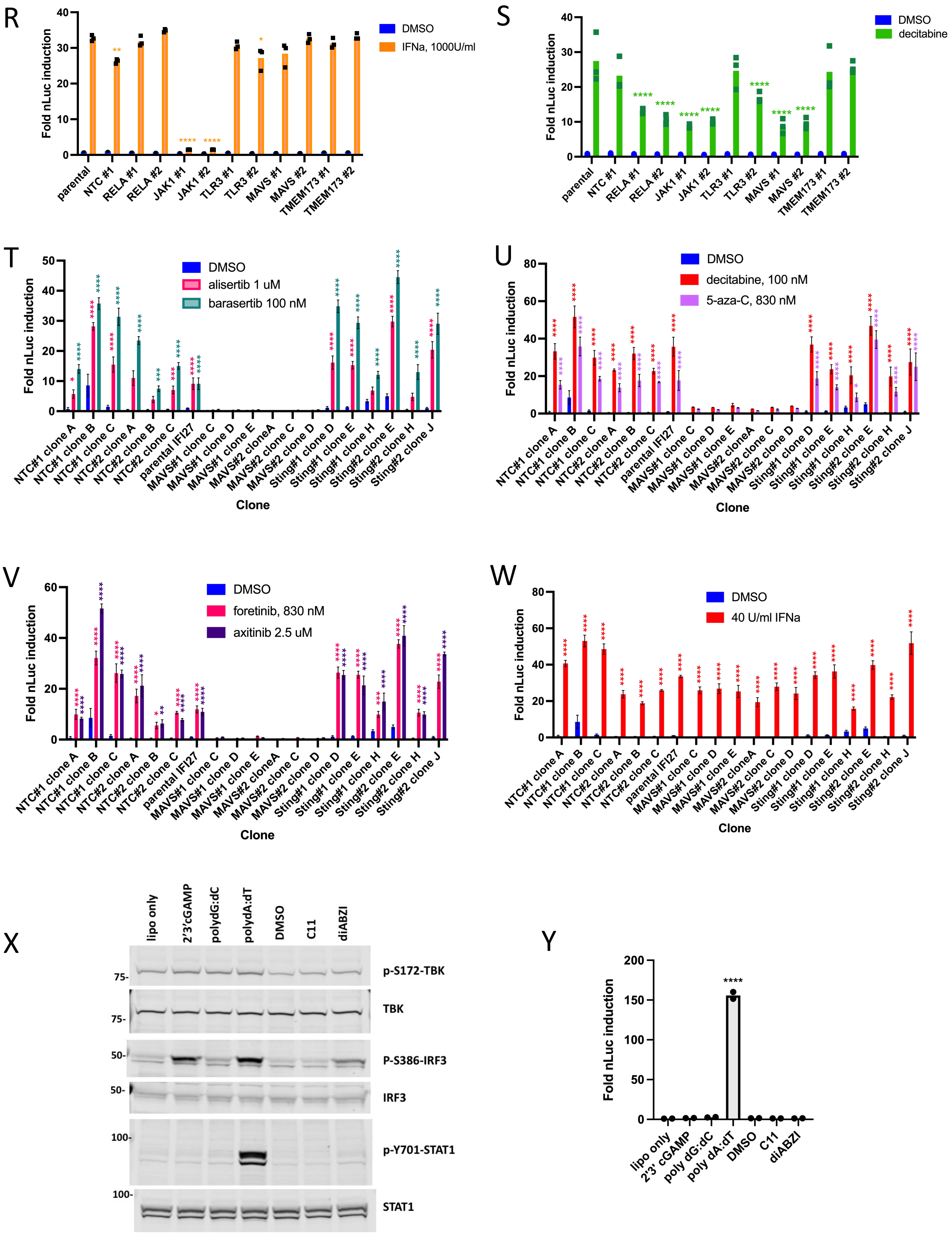

Supplemental Figure 4 cont'd

Supplemental Figure 4. IFN/PRR pathway analysis.

A) Validation of IFNLR1 knockout and IFNAR1 knockout *IFI27* reporter cell lines. WT, IFNAR1 KO, or IFNLR1 KO *IFI27* reporter cell lines were treated with the indicated dose of IFNβ (left panel) or IFNλ2 (right panel), then luciferase activity was assayed 24h later. Values are normalized to, and significance are expressed relative to, the no-interferon control.
B) Quantification of γ-H2AX immunofluorescence staining after alisertib or barasertib treatment of HCT116-*IFI27* cells.
C) Quantification of micronuclei after alisertib or barasertib treatment of HCT116-*IFI27* cells.
D) Immunofluorescence images of DMSO-treated HCT116-IFI27 cells stained for γ-H2AX (left), or Hoechst 33342 (right). Merged images with yellow for γ-H2AX and blue for Hoechst (center).
E) Same as D, but cells are treated with 1 uM alisertib.
F) Same as D, but cells are treated with 250 nM barasertib.
G) Immunofluorescence images of HCT116-IFI27 cells treated with 250 nM barasertib then stained for γ-H2AX (left), or Hoechst 33342 (right). Merged images with yellow for γ-H2AX and blue for Hoechst (center).
H) Quantification of γ-H2AX immunofluorescence staining after alisertib or barasertib treatment of HT-29 cells.
I) Same as D but with HT29 cells
J) Same as E but with HT29 cells.
K) Same as F but with HT29 cells
L) Validation of JAK1 KO lines. The *IFI27* reporter cell line was infected with lentiviruses expressing non-targeting control or JAK1- targeting guides (Supplemental Table 1), and protein extracts were prepared from stably selected pooled cell lines and blotted for JAK1 expression.
M) Validation of RELA/p65 KO lines. Same as L) but with RELA/p65.
N) Validation of IRF3 KO lines. Same as L) but with IRF3.
O) Validation of MAVS KO lines. Same as L) but with MAVS.
P) Validation of STING KO lines. Same as L) but with STING, and the cell lines were treated or not for 21h with 1000U/ml IFNα prior to harvest.
Q) Validation of TLR3 KO lines. Same as P) but with TLR3.
R) IFN induction by IFN depends on JAK1. HCT116-*IFI27* reporter cell lines with CRISPR KO of the indicated genes were treated for 5 days with 1000U/ml IFNα, then nanoluciferase activity measured. Significance is shown for each cell line vs the parental control for each treatment.
S) Reporter activation by DNMTi is partially dependent on JAK1, RELA, and MAVS, but independent of TLR3 and STING. HCT116-*IFI27* reporter cell lines with CRISPR KO of the indicated genes were treated for 5 days with 280 nM decitabine, then nanoluciferase activity measured. Significance is shown for each cell line vs the parental control for each treatment.

T) Reporter activation of single cell-derived clones in the reporter in response to AURKi depends on MAVS but not STING. Cells from the NTC control guide transfected population, MAVS KO population, or STING KO population, were subjected to a round of single cell cloning and expansion. Clones were then treated with the indicated doses of alisertib, barasertib, or vehicle DMSO and luciferase was assayed 5 days later. Values in T)-W) are normalized to the value of NTC#1 clone A with DMSO treatment, significance is shown each treatment vs. DMSO for each clone.

U) As in T), but measuring the response to decitabine or 5-azacytidine.

V) As in T), but measuring the response to foretinib or axitinib.

W) Lack of response to AURKi by MAVS KO clones is not due to a defect in IFN signaling. Clones from T) were treated or not with 40 U/ml IFNα, and luciferase assayed 24h later.

X) HCT116-*IFI27* reporter line does not phosphorylate STAT1 in response to agonists of STING signaling. Cells in 6 well plates were transfected with lipofectamine without or with 50 ng cGAMP, 250 ng poly dG:dC, or 250 ng poly dA:dT; alternatively cells were treated by addition of DMSO, 100 μM C11, or 10 μM diABZI STING agonists. After 26h lysates were prepared and blotted with the indicated antibodies.

Y) HCT116-*IFI27* reporter line does not activate IFN in response to agonists of STING signaling. Supernatants from cells in X) were assayed for luciferase activity.
