# Supplementary material for: Inhibition of Aurora Kinase Induces Endogenous Retroelements to Induce a Type I/III IFN Response via RIG-I
Source: Cancer Res Commun. 2024 Feb 26;4(2):540–55. doi: 10.1158/2767-9764.CRC-23-0432 (PMC10896070; doi:10.1158/2767-9764.CRC-23-0432)
Supplement: Supplemental Figure 5 — Aurora kinase inhibition does not affect genomic DNA CpG methylation. [file crc-23-0432-s13.pdf]

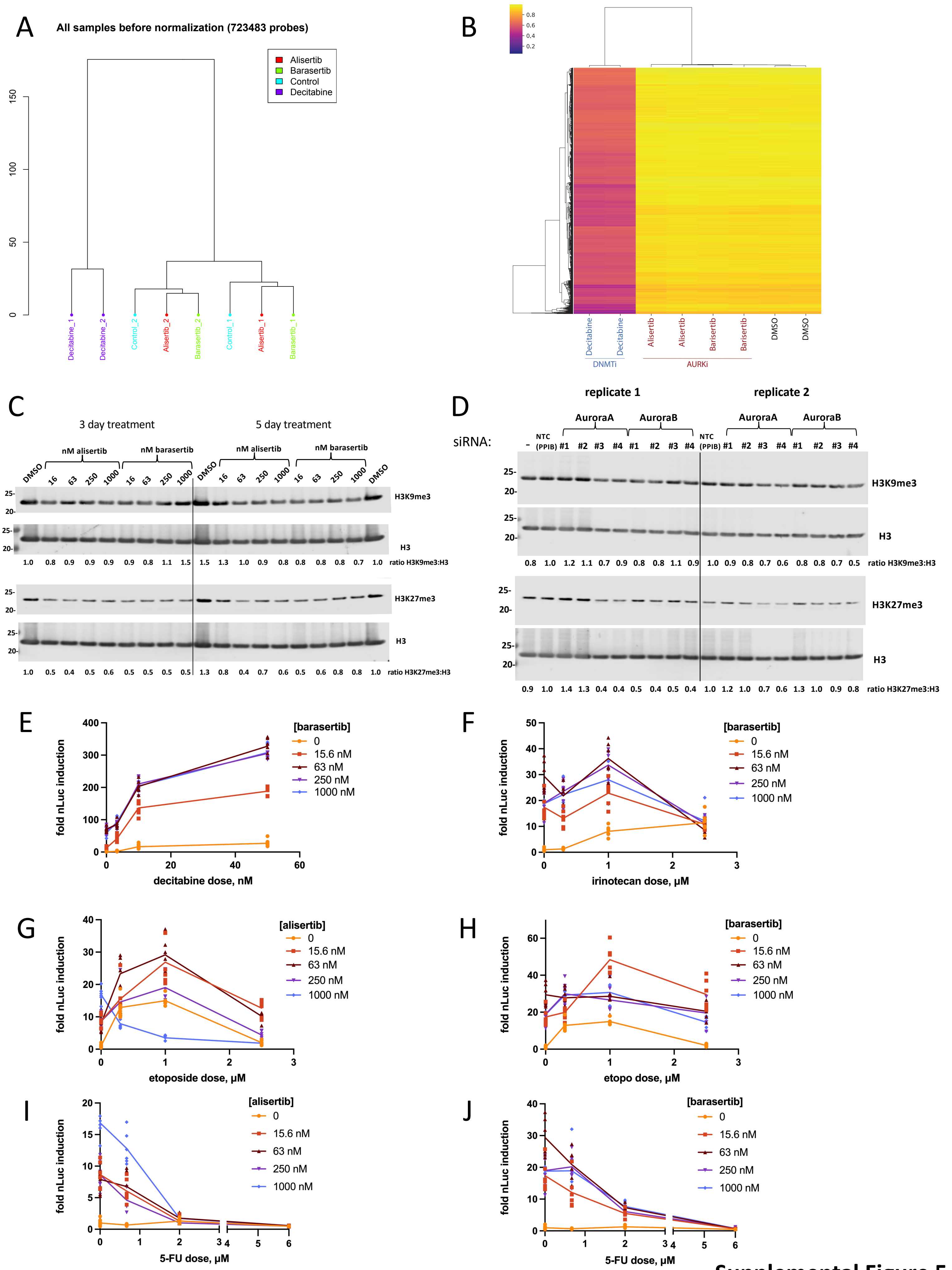


**Supplemental Figure 5**

**Supplemental Figure 5. Aurora kinase inhibition does not affect genomic DNA CpG methylation.**

A) Dendrogram of pre-normalization samples from ChAMP.QC. All CpGs were used directly to calculate distance matrix.
B) Heatmap of PBC-normalized methylation values of the top 10,000 differentially methylated probes (sorted by p-value in decitabine condition) across all conditions. No differentially methylated probes were detected in alisertib or barisertib conditions relative to control.
C) Western blot analysis of global H3K9me3 and H3K27me3 levels from cells treated with AURKi. Cells as in Figure 2D were treated for 3 or 5 days with the indicated doses of alisertib or barasertib. The resulting H3K9me3 or H3K27me3 signals were normalized to the respective total H3 signal intensity, and values expressed as fold change relative to the DMSO control for each time point. For the 5 day data, which has 2 independent DMSO samples, the value of the 2nd DMSO control at the far right was used as the reference value.
D) Western blot analysis of global H3K9me3 and H3K27me3 levels from cells with siRNA knockdown of Aurora A or Aurora B. As in Figure 2H, but blotted for H3K9me3, H3K27me3, or total H3. The resulting H3K9me3 or H3K27me3 signals were normalized to the respective total H3 signal, and values expressed as fold change relative to the PPIB siRNA transfected control.
E) Synergistic interaction between AURKi and DNTMi for IFN induction. HCT116-*IFI27* cells were treated for 5 days with the indicated doses of alisertib and/or decitabine, then reporter luciferase activity was measured.
F) As in C) but with alisertib and irinotecan. Synergy not observed.
G) As in C) but with alisertib and etoposide.
H) As in C) but with barasertib and etoposide.
I) As in C) but with alisertib and 5-FU.
J) As in C) but with barasertib and 5-FU.
