# Supplementary material for: Inhibition of Aurora Kinase Induces Endogenous Retroelements to Induce a Type I/III IFN Response via RIG-I
Source: Cancer Res Commun. 2024 Feb 26;4(2):540–55. doi: 10.1158/2767-9764.CRC-23-0432 (PMC10896070; doi:10.1158/2767-9764.CRC-23-0432)
Supplement: Supplemental Figure 6 — Characterization of CT26 Sting KO lines. [file crc-23-0432-s14.pdf]

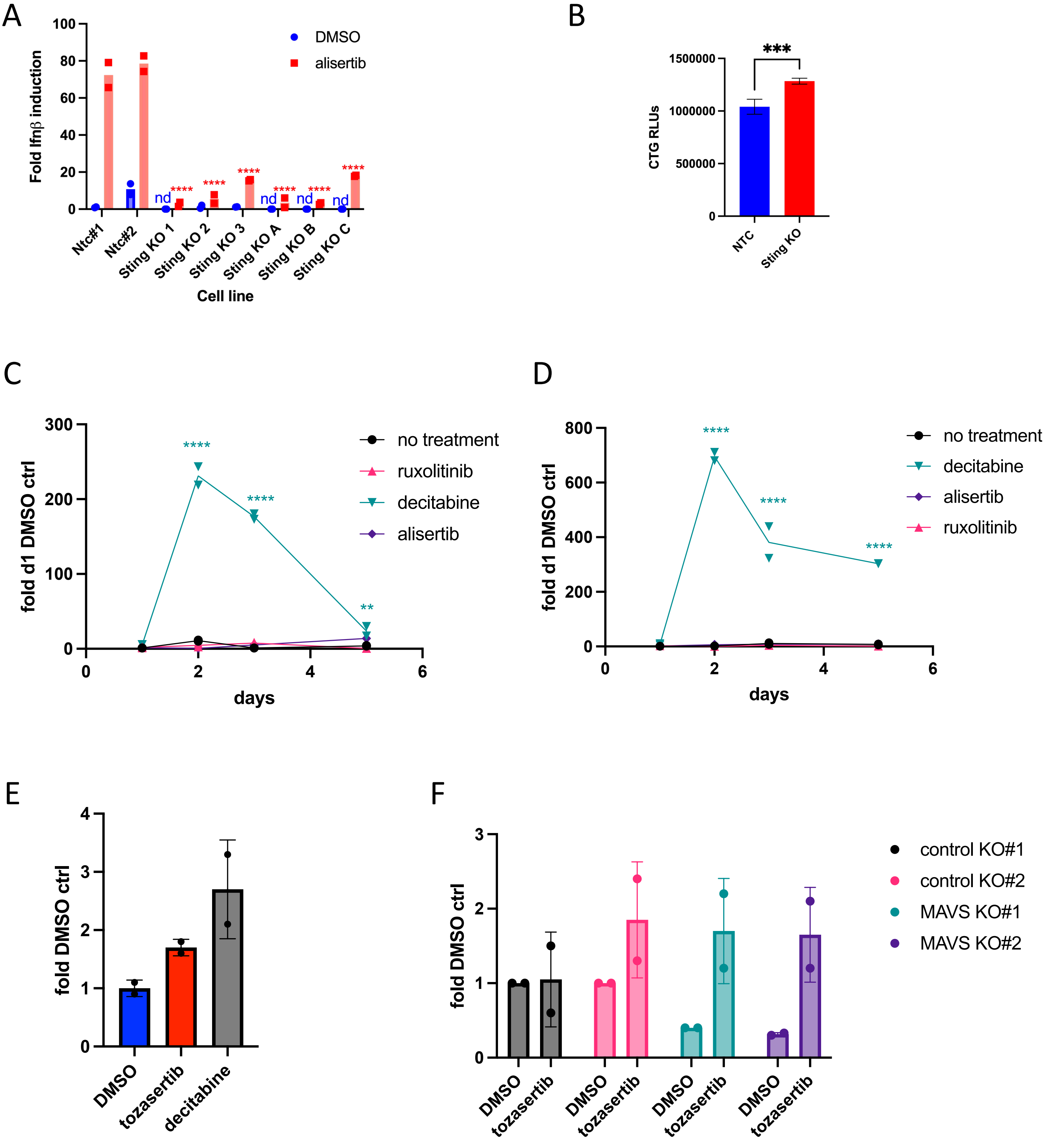

Supplemental Figure 6

Supplemental Figure 6. Characterization of CT26 Sting KO lines.

A) Single cell clones with Sting KO have deficient *Ifnβ* induction after alisertib treatment. QPCR for *Ifnβ* from CT26 control or Sting KO clones described in Figure 6H/I/J. Significance is shown comparing Ntc#1 vs each genotype for alisertib treatment. Four out of 6 Sting KO clones had no detectable amplication of *Ifnβ* ("n.d.") without alisertib treatment.

B) Sting loss does not impede growth in vitro. Cells used for the tumor inoculations for Figure 6H were seeded at 2000 cells per well of a 96 well plate and allowed to grow for 72h, the cell titer-glo viability was assayed. Significance testing indicates KO cells grow slightly faster than Ntc controls in vitro.

C) No induction of Ifn response to AURKi in 4T1 cells in vitro. Cells were treated with DMSO, 10 nM decitabine, 1 μM tozasertib, or 10 μM ruxolitinib for the indicated times, then RNA prepared and analyzed by Taqman for Ifnβ gene expression. Cells responded robustly to decitabine but not to other treatments.

D) No induction of Ifn response to AURKi in B16F10 cells in vitro. As in C, but with B16F10 cells.

E) No induction of Ifn response to AURKi in MC38 cells in vitro. MC38 cells were treated for 5 days with DMSO, 1 μM tozasertib or 10 nM decitabine, then RNA was prepared and analyzed by Taqman for Ifnβ gene expression.

F) No induction of Ifn response to AURKi, nor role for MAVS in blocking Ifn induction, in LLC cells in vitro. LLC cells with control or MAVS KO were generated by RNP transfection, and transfected cell pools with >90% MAVS KO or control guides to Olf555 were treated for 5 days with DMSO or 1 μM tozasertib, then RNA was prepared and analyzed by Taqman for Ifnβ gene expression.
