# Supplementary material for: Inhibition of Aurora Kinase Induces Endogenous Retroelements to Induce a Type I/III IFN Response via RIG-I
Source: Cancer Res Commun. 2024 Feb 26;4(2):540–55. doi: 10.1158/2767-9764.CRC-23-0432 (PMC10896070; doi:10.1158/2767-9764.CRC-23-0432)
Supplement: Supplementary Table Legends [file crc-23-0432-s15.docx]

***SUPPLEMENTAL TABLE LEGENDS***

**Supplemental Table 1.** **Sequences of siRNAs, gRNAs, and antibodies used in this study.**

**Supplemental Table 2. RNAseq data from HCT116 cells treated 24h with 1000U/ml IFNα.**

Biological triplicate RNA samples with or without IFN treatment were analyzed using our in-house RNAseq platform. Differential expression, p values and q values are shown.

**Supplemental Table 3. RNAseq data from drug treatments depicted in Figure 3A.**

Biological duplicate RNA samples with or without drug treatment were analyzed using our in-house RNAseq platform. Differential expression, p values and q values are shown.

**Supplemental Table 4. RNAseq from CRC lines.**

RNAseq analysis of LoVo, Ls174T, HT29, SW480, SW1417, or Ls123 cells were treated with either 1 μM alisertib, 100 nM decitabine, or DMSO for 5 days. Differential expression, p values and q values are shown.

**Supplemental Table 5. RNAseq from MAVS KO study.**

RNAseq analysis of single cell control or MAVS KO clones treated with 1 μM alisertib or DMSO for 5 days. Contrasts shown are DMSO vs alisertib for each clone, or each alisertib-treated control vs each alisertib-treated MAVS KO clone.

**Supplemental Table 6. ICE analysis of various CRISPR KO lines used in this study.**

Cell lines transfected with Cas9-sgRNA RNPs were evaluated for % knockout by PCR amplification of the region of targeted genomic DNA, Sanger sequencing and analysis of sequencing data using the ICE tool available at Synthego.

**Supplemental Table 7. TEtranscripts analysis results from RNAseq data from Figure 3A.**

**Supplemental Table 8. Chromatin modifier gene expression data in CRC lines after alisertib treatment.** RNAseq data from CRC lines that induced an IFN response after alisertib treatment were drawn from studies shown in Supplemental Tables 3 and 4, and analyzed for expression of chromatin modifiers.
